# Developing Novel Plant-Based Probiotic Beverages: A Study on Viability and Physicochemical and Sensory Stability

**DOI:** 10.3390/foods14122148

**Published:** 2025-06-19

**Authors:** Concetta Condurso, Maria Merlino, Anthea Miller, Ambra Rita Di Rosa, Francesca Accetta, Michelangelo Leonardi, Nicola Cicero, Teresa Gervasi

**Affiliations:** 1 Department of Veterinary Sciences, University of Messina, 98168 Messina, Italy; anthea.miller@studenti.unime.it (A.M.); ambra.dirosa@unime.it (A.R.D.R.); francesca.accetta@studenti.unime.it (F.A.); 2 Department of Biomedical and Dental Sciences and Morphofunctional Imaging, University of Messina, 98168 Messina, Italy; michelangelo.leonardi@unime.it (M.L.); nicola.cicero@unime.it (N.C.); teresa.gervasi@unime.it (T.G.); 3 Science4life S.R.L.—Start Up, University of Messina, 98168 Messina, Italy

**Keywords:** probiotic *Lactobacillus* spp., primary shelf life, secondary shelf life, E-Eyes, probiotic viability, volatile aroma profile

## Abstract

Consumer demand for plant-based functional foods, especially probiotic beverages, has increased due to their health benefits and suitability as dairy-free alternatives. This study assessed, through a factorial combination, the stability of plant-based extracts (avocado, ginger, and tropical) individually inoculated with three commercial *Lactobacillus* strains (*L. casei*, *L. plantarum*, *L. reuteri*) and stored under refrigerated conditions during both primary (PSL) and secondary shelf life (SSL). Product shelf life was defined by probiotic viability, considering the functional threshold (≥6 log CFU/mL), which was maintained across all formulations throughout the storage period. Physicochemical parameters, including pH, titratable acidity, and colour, as well as volatile profile, remained stable, with only minor variations depending on the matrix and bacterial strain. Sensory evaluations (triangle and acceptability tests) confirmed that the probiotic juices were acceptable to consumers. Overall, the results demonstrate the feasibility of producing non-fermented, plant-based probiotic beverages that retain their functional properties and meet consumer sensory expectations, offering a promising alternative for vegan and lactose-intolerant individuals.

## 1. Introduction

In recent years, there has been a rise in consumer interest toward plant-based functional foods—especially probiotic-fortified beverages—driven by their perceived health benefits and suitability for no-dairy products [1,2].

Traditionally, probiotic foods on the market have been dairy based, but growing awareness of milk allergenicity, lactose intolerance, and the rise in vegan diets have driven the demand for non-dairy alternatives [2,3,4].

In response, fruits and vegetables are increasingly being explored as novel matrices for probiotic delivery, offering an attractive tool that aligns with “clean label” and vegan product tendencies [2]. Fruit and vegetable beverages represent ideal carriers for probiotics, as they are naturally rich in vitamins, antioxidants, and other phytochemical compounds [5].

Probiotic products have been linked to improved gut health, immune function, and overall well-being, which is attractive from a public health perspective [1,3,5,6].

Fruit juices provide an excellent base for developing probiotic beverages due to their pleasing taste, which appeals to all age groups, and their perception as healthy and refreshing options. The use of probiotics for the fortification of vegetable juices paves the way for a new class of functional beverage that combines the health advantages of probiotics with the typical nutritional benefits of fruits and vegetables [1,7].

Nonetheless, formulating stable, non-fermented probiotic beverages presents several challenges. Probiotic viability is a crucial concern. To be effective, probiotic foods typically require a minimum concentration of 10⁶ CFU/mL or a daily intake of 10⁸–10^11^ [8], and live cell count must be maintained throughout the shelf life of the product [5]. However, the acidic pH and high organic acid content of many fruit juices can impose acid stress on probiotic bacteria, leading to gradual viability loss during refrigerated storage [2,5]. Additionally, other factors, like high sugar levels, salt concentration, metabolic products, dissolved oxygen, and redox potential, can negatively impact probiotic cells in juice environments [5]. Furthermore, another key challenge is sensory and physical stability. Probiotic microorganisms may impact flavour, aroma, or clarity over time [1,5].

To address these issues, recent research has focused on technological strategies to improve probiotic viability in non-dairy beverages, including strain selection (such as selecting inherently robust and acid-tolerant strains of *Lactobacillus*), fortifying the juice with prebiotic fibres or polyphenols, and microencapsulation of probiotic cells, which have all shown success in mitigating cell loss during storage [1,9]. However, before introducing additional interventions, it was deemed important to first assess the intrinsic ability of the selected probiotic strains to survive in realistic, commercially relevant, plant-based matrices.

The genus *Lactobacillus* was selected for its well-documented use as a widely studied group of probiotic microorganisms, particularly in the fortification of non-dairy food matrices such as fruit juice. This genus is favoured for its established safety profile, health-promoting properties, and its notable adaptability to complex food matrices [3,10]. In the current study, blended fruit and fruit–vegetable extracts were selected based on two criteria: (i) their growing popularity and consumer acceptance as non-diary functional beverage bases, and (ii) the potential synergistic effect of combining different fruits and vegetables in terms of nutritional composition, antioxidant capacity, and sensory balance. These matrices, made up of 100% blended fruits or vegetable–fruits, were fortified with three commercial probiotic *Lactobacillus* strains, each utilised as a single-strain inoculum, to assess their viability and stability over time without the use of protective technologies. This objective is rooted in the growing interest in using natural ingredients to enhance the functionality and stability of probiotics, particularly in the context of clean-label, health-conscious food and supplement products. Furthermore, the research aimed to evaluate the physiochemical stability and the maintenance of juice sensory quality during both primary shelf life (PSL) and secondary shelf life (SSL).

## 2. Materials and Methods

### 2.1. Plant-Based Extracts

The following commercially available blended fruit and fruit–vegetable extracts were used: (1) avocado extract, composed of 47% apple, 25% pear, 20% avocado, 8% spinach; (2) ginger extract, composed of 93% apple, 7% ginger; and (3) tropical extract, composed of 42% apple, 35% mango, 10% pineapple, 8% banana, and 5% passion fruit. All the extracts were pure (no added water or sugar) and free of preservatives, flavourings, and colourants. The extracts were packed in 250 mL PET bottles under nitrogen, pasteurised by high-pressure processing (HPP), and then stored at 4 ± 1 °C for the entire storage period. The extracts were kindly supplied by an Italian fruit and vegetable processing company.

These products were selected as representative of real-market substrate for functional beverage development.

### 2.2. Bacterial Strain and Growth Conditions

The probiotic strains used to fortify the selected extracts included the following probiotic *Lactobacillus* strains: *L. reuteri* DSM 17938 and *L. plantarum* LP09 obtained from a commercial suspension; and *L. casei* isolated from a commercial drinking yoghurt.

Strains were individually propagated in De Man, Rogosa, and Sharpe (MRS, Oxoid, Milan, Italy) medium at 37 ± 2 °C for 24 h.

### 2.3. Experimental Design

The experimental design of the study involved preparing 12 formulations (3 extracts × 3 strains, plus 3 non-inoculated control samples). Measurements were taken at five different time intervals throughout the PSL (i.e., before package opening) from production up to 40 days, and at four time intervals during the SSL (i.e., after package opening) up to 4 days (Table 1). All analyses were conducted in triplicate across two separate experiments.

### 2.4. Inocula Preparation and Fortification of Plant-Based Extracts

To be used as fortifiers, lactic acid bacteria were cultivated until they reached late exponential growth (approximately 16 h). The cells were then harvested by centrifugation (8000× *g*, 4 °C, 10 min), washed twice in 50 mM phosphate buffer (PBS, 4 °C, pH 7.0), and resuspended in sterile tap water to achieve a final cell density of about 9 log colony-forming units (CFUs) per mL of solution. Each strain was then individually inoculated into the fruit extracts to obtain the fortified products, with an initial concentration adjusted to approximately 10^6^–10^7^ CFU/mL. This range reflects the commonly accepted probiotic levels used in functional food formulations to ensure potential health benefits upon consumption.

The inoculation with the three selected probiotic strains was carried out under conditions that allowed for the maintenance of existing modified atmosphere packaging.

After the inoculation procedure, the samples were rapidly cooled in an ice-water bath and stored at 4 ± 1 °C and analysed at different intervals through PSL and SSL. Unfortified extracts served as controls.

### 2.5. Microbiological Analyses

The fruit and fruit–vegetable extracts were microbiologically characterised by counting different classes of microorganisms. A 25 mL sample was diluted with 225 mL of PBS (pH = 7.4), then serial dilutions were performed, and the samples were spread aseptically over the media plates.

For mesophilic counts, samples were spread over plate count agar (PCA, Oxoid, Milan, Italy) and incubated at 30 ± 2 h °C for 48 ± 2 h. Yeasts and moulds were evaluated using malt extract agar (MEA, Oxoid, Milan, Italy) with 10% lactic acid after incubation at 30 ± 2 °C for 72 ± 2 h.

Enterobacteriaceae were investigated using Violet Red Bile Glucose Agar (VRBG, Oxoid, Milan, Italy) with an incubation period of 48 ± 2 h at 37 ± 2 °C.

Coliform microorganisms were enumerated using the Most Probable Number (MPN) system with Lauril Triptose Broth (LTB, Oxoid, Milan, Italy) for 48 ± 2 h at 37 ± 2 °C, followed by confirmation with Brilliant Green Bile 2% (BGB, Oxoid, Milan, Italy) after 48 ± 2 h for total coliforms and Green Bile 2% and Tryptone Water (TW, Oxoid, Milan, Italy) after 24 ± 2 h at 44 ± 2 °C for faecal coliforms. All media were autoclaved at 121 °C for 15 min before use, except for VRBGA, which was heated to boiling point without being autoclaved.

### 2.6. Viability of Lactic Acid Bacteria Strains in Plant-Based Extracts During Storage

Immediately after inoculation and at fixed intervals during PSL and SSL, a 100 μL aliquot of each fortified extract was serially diluted in sterile saline solution and plated on MRS agar using the micro-drop technique [11]. Viable cell counts were determined after a 48 h incubation period at 37 ± 2 °C on MRS agar (Oxoid, Milan, Italy), and results were expressed as log CFU/mL.

### 2.7. Physicochemical Parameters 

#### 2.7.1. pH

The pH of the extract samples was measured using a benchtop XS pH 8 standard pH metre (XS Instruments, Carpi, Italy), equipped with a Hamilton Polyte Lab electrode (Hamilton, Bonaduz, Switzerland), along with a magnetic stirrer, and a temperature probe. The electrode and temperature probe were inserted directly into the extract. Before the measurement, each extract sample was left to equilibrate to room temperature.

#### 2.7.2. Titratable Acidity

The titratable acidity (TA) was determined through titration with 0.1000 N NaOH, using a phenolphthalein (1% ethanolic solution) as an indicator. For the determination, a 10 mL aliquot of each extract was diluted to 35 mL with carbonate-free double-distilled water. Due to the extract colourations, the endpoint of the titration was confirmed by measuring the pH of the mixture (endpoint to pH = 8.3) using the instrumentation mentioned above. The results were expressed as g of citric acid/100 mL of extract.

#### 2.7.3. Colour

Colour monitoring of the fruit and fruit–vegetable extracts was performed using a commercial E-Eye (Iris Visual Analyzer 400 system, Alpha M.O.S. Toulouse, France), equipped with a high-resolution camera and dual illumination (top and bottom), capable of capturing up to 16 million colours (RGB colour code). To ensure optimal image acquisition quality, bottom lighting was used to provide uniform light without reflections. Additionally, a white background was used to eliminate any potential distortions or visual interferences. Each extract sample was placed on the platform of the measurement chamber, inside a 6 cm diameter Petri dish. For each extract sample, three photos were taken, ensuring repeatability and greater reliability of the collected data. After image acquisition, the visual data underwent an optimisation process, which included background removal and enhancement of overall image quality. Once the method was validated, it was applied uniformly to all images. The thresholds values used were as follows: R (255, 254), G (255, 255), B (255, 185) [12].

### 2.8. Volatile Fraction

The headspace Solid-Phase Microextraction-Gas Chromatography/Mass Spectrometry (SPME-GC/MS) technique was used for the analysis of the volatile fraction of both probiotic and control fruit and fruit–vegetable extracts. Specifically, an 18 mL aliquot of either avocado or tropical extracts was transferred to a 40 mL vial, added with 6 g of NaCl (ACS reagent, Merck Life Science S.R.L., Milan, Italy), and sealed with a “mininert” valve (Supelco, Bellefonte, PA, USA). For ginger extracts, the samples needed to be diluted with distilled water at a ratio of 1:8 (extract to water) *v*/*v*.

The vials were placed in a water bath and allowed to thermally equilibrate at 35 °C for 30 min. For volatile sampling, a DVB/CAR/PDMS fibre, with a film thickness of 50/30 μm (Supelco, Bellefonte, PA, USA), was exposed to the headspace of the sample for 20 min. During this time, the sample was maintained at 35 °C and subjected to continuous magnetic stirring.

The fibre was then inserted into the injector port of a Shimadzu GC/ TQMS (Shimazu GC 2010 Plus/-TQMS 8040, Shimadzu Italia s.r.l., Milan, Italy) for desorption of volatile compounds and subsequent chromatographic separation on a CP-WAX-52-CB capillary column, 60 m × 0.25 mm i.d. × 0.25 µm film thickness (Agilent S.p.A., Turin, Italy).

The chromatographic conditions were as follows: injection temperature, 260 °C; injection mode, splitless; oven temperature, 45 °C for 2 min, increased to 210 °C at 3 °C/min, and to 260 °C at 20 °C/min; carrier gas, helium at a constant flow rate of 1 mL/min; transfer line temperature, 250 °C; acquisition range, 40 to 400 m/z; scan speed, 1250 amu/s.

Volatile compounds were identified using the mass spectral data, the NIST’ 14 (NIST/EPA/NIH Mass Spectra Library, version 2.0, Gaithersburg, MD, USA) and FFNSC 3.0 databases (Shimadzu, Kyoto, Japan), linear retention indices (LRIs) calculated according to the Van Den Dool and Kratz equation, and injection of available standards [13]. Quantitative results were expressed as a percentage of the total peak area in the total ion current (TIC) chromatogram [13].

### 2.9. Sensorial Analyses

Sensory evaluation was performed by an untrained consumer panel consisting of 78 participants (40 females and 38 males) aged between 20 and 38 years. They were randomly selected among non-smoking students and staff of the University of Messina who regularly consume fruit and fruit–vegetable extracts. All participants were volunteers and signed an informed consent form in accordance with the principles of the Declaration of Helsinki. The sensory evaluation included a triangle test (performed based on ISO 4120:2021) [14], and a preference test and acceptance test performed according to ISO 11136:2014 [15]. The triangle and preference tests were conducted in the same session on extracts during the SSL. The acceptance test was conducted on extracts during the PSL. Samples of about 40 mL were served in clear shot glasses at a serving temperature of 8 ± 2 °C. Plain water was provided for the judges to rinse their mouths between each sample tasting. Evaluations took place in individual booths illuminated with white light.

#### 2.9.1. Triangle Test

Each judge received a set of three tasting samples, two of which were the same and one different, and was asked to indicate which was the odd sample. After identifying the odd sample, each judge was required to express which sample was preferred. The samples were coded using a three-digit number so that the judge could not recognise the samples themselves.

The samples analysed were the probiotic avocado, ginger, and tropical extracts at different times (days) after opening, specifically 1 day (T1) and 4 days (T4). The T1 and T4 samples were evaluated in two different sessions and, each time, compared with a sample opened at that time (T0).

Test preparation consisted of distributing the two samples to compare, T0 and Tx, according to a scheme in which the possible combinations were

T0TxTx − TxT0Tx − T0T0Tx − TxT0T0 − T0TxT0 − TxTxT0 where x was equal to 1 or 4

The samples were equally distributed within the panel such that some judges received two shot glasses with sample T0 and one with sample Tx, and an equal number of judges, conversely, received two shot glasses with sample Tx and one with sample T0. Before the start of each tasting session, judges were instructed on how to perform the test and on the tasting sequence (from left to right).

#### 2.9.2. Acceptability Test

The sensory acceptability of probiotic fruit and fruit–vegetable extracts was evaluated across three different tasting sessions. In each session, judges assessed the control and probiotic samples of the three fruit extracts at the end of the PSL and the beginning of the SSL. Within each session, tasting of one type of extract was followed by a 10 min break, and participants used water to rinse their mouths between samples. The order of sample presentation was randomised across judges and sessions.

Consumers were asked to rate the appearance, odour, flavour, taste and aftertaste of the extracts according to a nine-point hedonic scale (1 = extremely unpleasant; 2 = very unpleasant; 3 = unpleasant; 4 = slightly unpleasant; 5 = neither pleasant nor unpleasant; 6 = slightly pleasant; 7 = pleasant; 8 = very pleasant; 9 = extremely pleasant). The average of all sensory descriptors was then used to calculate the overall acceptability of the extracts.

### 2.10. Statistical Analysis

XLStat software, version 2024.1 (Addinsoft, New York, NY, USA) was used. Two-way ANOVA and Tukey’s multiple range test, conducted at a 95% confidence level, were used on microbiological, chemical, and sensory data to identify significant differences among the samples.

## 3. Results

### 3.1. Chemical Composition of Fruit and Fruit–Vegetable Extracts

Table 2 reports the proximate composition and nutritional value of the fruit and fruit–vegetable extracts. As expected, carbohydrates, almost entirely made up of simple sugars, are the main constituents.

The three extracts did not show any significant differences in their sugar content. However, the ginger extract had the highest carbohydrate percentage, largely due to its statistically significant higher fibre amount. Conversely, the tropical extract exhibited the lowest fibre amount. Avocado extract was characterised by a high content of total lipid, which was around six times greater than that of the other two extracts. This lipid content is related to the high fat content of avocado pulp, which is reported to range from 8.4% to 26.7% depending on the avocado physiological maturity and variety [16]. In addition to total fat, the avocado extract also contained statistically significant higher levels of protein, ash, and sodium chloride compared to the other extracts. In terms of phytochemicals, all the extracts contained noteworthy amounts of total phenolics (TPC) and total flavonoids (TFC), with TPC ranging from 27.5 to 45.0 mg AGE per 100 mL and TFC ranging from 26.7 to 49.3 mg catechin per 100 mL (Table 3).

The avocado extract had the highest TPC but the lowest TFC, while the tropical extract showed the highest levels of both TPC and TFC. These extracts consisted entirely of either 100% fruit or a fruit–vegetable mix, and they did not undergo any thermal treatments; this suggests that their phytochemical content is influenced by the natural phytochemical levels present in the fruits and vegetables used for their formulation [17,18].

### 3.2. Microbiological Counts

The microbiological analysis of the products was conducted on the three extracts to ensure that the hygienic-sanitary criteria guaranteed by the marketing company were not altered by the fortification processes. Throughout all assessments, the complete absence of coliform and Enterobacteria microorganisms was recorded. Similarly, the microbial loads related to aerobic microorganisms and acidophiles were below those commonly considered critical for the hygienic-sanitary acceptability throughout the entire storage period of both PSL and SSL.

### 3.3. Probiotic Cell Viability

In this study, three non-diary extract formulations were evaluated as potential carriers for delivering and sustaining the viability of three commercially available lactic acid bacterial strains.

*Lactobacillus* spp. was selected as representing one of the most extensively studied genera of probiotic microorganisms used for fruit juice fortification. This genus is particularly favoured due to its well-documented safety, health-promoting properties, and adaptability to food matrices, making it a suitable candidate for functional food applications [3,19]. 

To ensure the survival and activity of probiotics when incorporated into fruit juices, several studies have been conducted on the composition of the food matrices, as well as on various protection techniques. Probiotic stability in fruit juices can be improved by supplementing the formulation with prebiotics [20]. Microencapsulation is widely recognised for its effectiveness in protecting probiotics in fruit juices enhancing bacterial survival through the gastrointestinal tract by shielding them from acidic and oxidative conditions [20,21]. Spray-drying is efficient in stabilising probiotics; nevertheless, viability may be negatively impacted by the heating [22,23]. Freeze-drying maintains high probiotic survival rates, but protective agents are necessary to prevent cell damage [24].

This study focused on incorporating probiotics in their free form, a strategy previously reported to be effective in maintaining microbial viability in similar applications [2]. The concentration of probiotics added to the extracts was about 10^7^ CFU/mL, aligning with the FAO/WHO guidelines, which recommend that probiotic products maintain a minimum of 10^6^ to 10^7^ CFU/mL throughout their shelf life [2].

**Figure 1:**
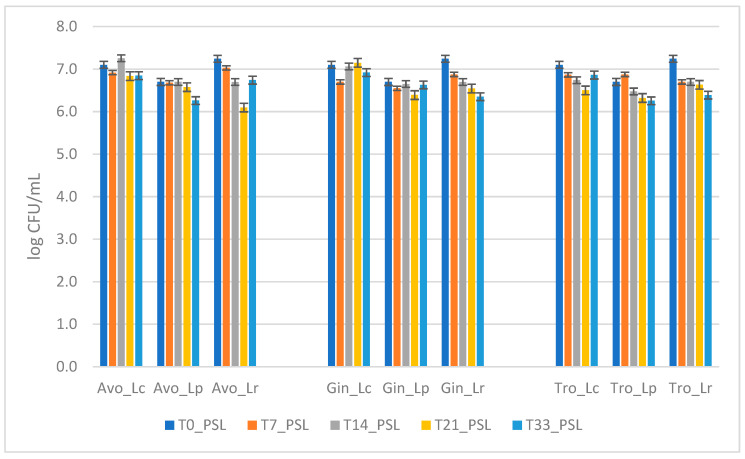
A comparison of microorganism survival in the three extracts during primary shelf life. The graph shows the mean survival of probiotics in each extract over time. The error bars represent the standard deviation. Two-way Anova did not reveal any statistically significant differences based on strain, storage time, or strain × storage time interaction.

During the PSL, no statistically significant variations were observed in the viable probiotic cells, based on strain, storage time, or strain × storage time interaction. Slight, and not significant (*p*> 0.05), reductions of 0.50, 0.89, and 0.85 log CFU/mL were observed for the microorganism *L. reuteri* at the end of the primary shelf life in avocado, ginger, and tropical extracts, respectively (Figure 1). However, the viability of all the microorganisms remained above 6 log CFU/mL in all tested extracts throughout the storage period, confirming the probiotic potential of the beverages.

**Figure 2:**
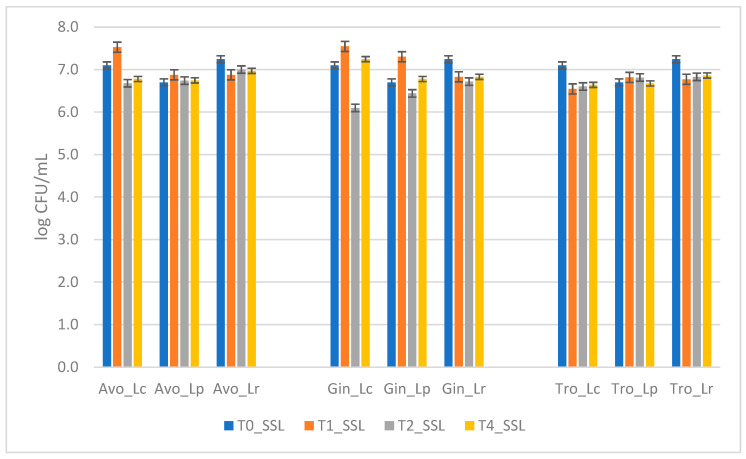
A comparison of microorganism survival in the three extracts during secondary shelf life. The graph shows the mean survival of probiotics in each extract over time. The error bars represent the standard deviation. Two-way Anova did not reveal any statistically significant differences based on strain, storage time, or strain × storage time interaction.

At the end of the SSL, reductions below 0.5 log CFU/mL were observed, confirming the stability of the probiotic in the extracts. Specifically, reductions of 0.41 log CFU/mL for *L. reuteri* in ginger extract, 0.32 log CFU/mL for *L. casei* in avocado extract, and 0.46 and 0.38 log CFU/mL for *L. casei* and *L. reuteri* in tropical extract, respectively, were recorded. The other observed decreases were under 0.3 log CFU/mL, and no reductions in the number of microorganisms below 6 log CFU/mL were observed (Figure 2). From two-way Anova, all these differences resulted to be not statistically significant (*p* < 0.05).

Interestingly, the stability observed in the primary shelf life continued into the secondary shelf life.

Numerous commercial and experimental products have been developed using pineapple, mango, carrot, beetroot, orange, and other tropical fruits as substrates for probiotics [25]. Probiotic viability in these matrices is highly strain-dependent and influenced by factors such as juice pH, organic acid profile, oxygen exposure, antimicrobial compounds, and processing methods [25]. These fruits are also rich in vitamins, minerals, dietary fibre, carbohydrates, bioactive compounds, and antioxidants, making them excellent and advantageous alternatives to dairy-based probiotic carriers. Generally, Lactobacilli remain viable above the probiotic threshold (~6 log CFU/mL) in low-phenolic, moderately acidic juices during refrigerated storage, with only minor population declines. However, it has been stated that in non-fermented probiotic fruit juices, the survival of *L. reuteri*, *L. casei*, and *L. plantarum* is highly strain- and matrix-dependent.

For example, *L. reuteri* DSM 20016 retained recommended counts (>10^6^–10^7^ CFU/mL) in pineapple, orange, and apple juices stored at 4 °C, whereas in an anthocyanin- and phenolic-rich berry juice, its viability dropped by 1 log within about 12 h due to the combined effects of low pH and phenolics [26].

Similarly, certain *L. casei* strains exhibit excellent stability in acidic juices: *L. casei* DN-114001 and *L. paracasei* NFBC43338 displayed good survival in orange and pineapple juices for up to 12 weeks at 4 °C, maintaining final counts above 6–7 log CFU/mL [27]. Conversely, *L. salivarius* strains UCC118 and UCC500 showed poor viability (<10^6^ CFU/mL) in the same matrices after only two weeks of storage [27].

Nualkaekul and Charalampopoulos [28] reported that *L. plantarum* NCIMB 8826 showed minimal losses (<0.4 log CFU/mL) over 6 weeks at 4 °C in orange, blackcurrant, and pineapple juices (pH ≈ 3.8), maintaining >7 log CFU/mL. In contrast, this strain declined rapidly in highly acidic and phenolic juices like cranberry and pomegranate, likely due to the presence of antimicrobial compounds such as phenolics.

Finally, *L. casei* showed poor survival in polyphenol-rich juices (e.g., raspberry or pomegranate), often falling below detectable levels after 4 weeks of storage. However, microencapsulation techniques improved its stability in these challenging matrices [29].

A previous study attributed the enhanced survival of *Lactobacillus plantarum* during refrigerated storage in malt-based extracts, as opposed to those derived from wheat and barley, to the higher residual sugar content present in malt. These fermentable sugars facilitate ATP synthesis, which plays a critical role in maintaining intracellular pH homeostasis and promoting cell viability under acidic conditions [30,31]. Our results are consistent with previous findings, as the sugar content in our formulations ranged between 9% and 10%, which likely contributed to the maintenance of probiotic viability throughout the storage period.

Fruits and vegetables are increasingly recognised as suitable carriers for probiotics thanks to their richness in vitamins, minerals, dietary fibre, carbohydrates, and antioxidants, making them excellent and advantageous alternatives to dairy-based matrices [30,31].

The production of functional plant-based extracts with improved probiotic stability represents an innovative approach for delivering beneficial bacteria through non-dairy food products. The interaction between the juice matrix, containing bioactive metabolites, phenolic compounds and antioxidants, and probiotics could contribute to gut health promotion [21,32,33]. The results obtained in this test are promising for the potential use of these juices as probiotic carriers.

### 3.4. Physicochemical Stability

#### 3.4.1. pH and Titratable Acidity

During the primary shelf life, the pH values of both the control and the probiotic extract samples remained stable. As shown in Table 4, no statistically significant differences were observed based on the strain, storage time, or their interaction (strain × storage time). The only exception was the avocado extracts, which exhibited a slight increase in pH values over the storage period, reaching the highest value after 21 days of refrigeration, regardless of whether probiotic bacteria were present.

Juice pH is an important indicator of the microbiological quality and safety of fruit juices. Fresh avocado and ginger extracts have a pH value of around 4, while the tropical extract has a pH of approximately 3.8. These values indicate that the selected fruit and fruit–vegetable extracts create an acidic environment, effectively inhibiting the growth of pathogenic organisms [34].

The stability of pH throughout the storage period is a clear sign of the efficacy of high-pressure processing in attaining microbiological stability. Indeed, pH decreases as the viable count of spoilage bacteria increases [35]. Many authors reported no significant modifications with storage period in the pH of fruit juice subjected to thermal pasteurisation, high pressure, or high intensity pulsed electric field treatments [36,37]. Conversely, the slight increase in the pH of avocado extract over time can be attributed to changes in the chemical or biochemical substances present in the juice, such as ascorbic acid degradation [38] or unsaturated fatty acid oxidation, as already reported [35,39].

Regarding the trend of pH values during the secondary shelf life, a pH decrease was observed in all fruit and fruit–vegetable extracts. After opening the juice packages, both sterility and modified atmosphere packaging (MAP) were compromised. This led to a moderate increase in the viable counts of aerobic microorganisms and acidophilic bacteria over the four days of refrigeration storage. Their metabolic activity may contribute to the pH reduction observed in all extracts.

Table 5 presents the titratable acidity (TA) of the fruit and fruit–vegetable extracts during both primary and secondary shelf life, under refrigerated storage conditions.

TA is expressed as a percentage of citric acid. A two-way ANOVA revealed significant differences in TA based on strain, storage duration, and their interaction, depending on the type of extract, across both shelf life phases. Referring to primary shelf life, the titratable acidity was significantly affected by storage time in avocado and tropical extracts. Conversely, no statistically significant differences were found in the TA of ginger extracts based on either strain, storage time, or their interaction. During the secondary shelf life, the titratable acidity was significantly affected by storage time in ginger extracts, by strain in tropical extracts, and by strain, storage time, and strain × storage time interaction in avocado extracts.

**Figure 3:**
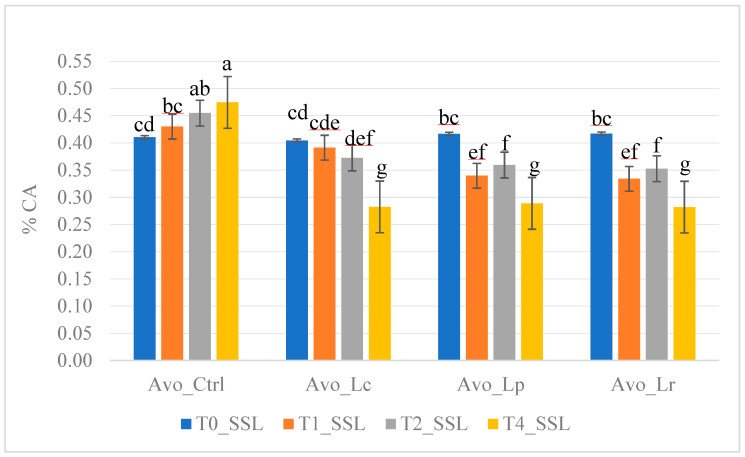
Effects of strain × storage time interaction on titratable acidity (g citric acid/100 mL) of avocado extracts during secondary shelf life under refrigerated storage conditions. Different letters (a–g) on the bars indicate significant differences by Turkey’s multiple range test at *p* < 0.05.

Before package opening, we observed a significant decrease in TA in avocado extracts at 7-day storage time (T7_PSL) and in tropical extracts at time zero (T0_PSL). After package opening, the three types of extracts exhibited different behaviour. Indeed, the TA of tropical extracts was influenced (*p* < 0.001) by the added strains, with the highest TA detected in the control sample and the lowest in the tropical extract fortified with *Lactobacillus plantarum* LP09. For ginger extracts, a significant difference was detected between the T2 and T1 samples during storage time. Finally, the TA of avocado extracts significantly varied based on the strain, storage time, and their interaction (Figure 3); as shown in Figure 3, the TA increased with storage time in the control sample (Avo_Ctrl), whereas it decreased in the avocado extracts fortified with probiotics.

Many studies have investigated the stability of pH and acidity in non-fermented probiotic juices during refrigerated storage, reporting inconsistent trends. For instance, Ding and Shah [40] observed a decrease in pH and an increase in acidity in orange and apple juices fortified with various strains of Lactic acid bacteria or Bifidobacteria over six weeks of refrigeration. Similarly, Miranda et al. [41] reported an acidity increment in orange juice after adding L. casei, though pH and TSS remained unchanged. Gumus et al. [42] noted a comparable trend in grape juice fortified with Lactobacillus acidophilus. Conversely, Kardooni et al. [43] reported no significant changes in pH or acidity during storage at 4 °C in orange juice supplemented with Lactobacillus acidophilus. These changes mainly result from the metabolism of probiotics. Indeed, even under refrigeration, the metabolic activity of probiotic bacteria cannot be entirely ruled out. As already demonstrated, the extent and direction of these variations are highly dependent on the specific combination of probiotic strains and the type of fruit juice used [44,45].

#### 3.4.2. Colour Profile

The evaluation of colour in probiotic fruit juices represents a crucial step in assessing product quality, as colour is a key determinant of consumer perception and may reflect attributes such as freshness or the occurrence of potential alterations. In this context, the E-Eye system was employed, which enables the acquisition of highly detailed and precise images.

**Figure 4:**
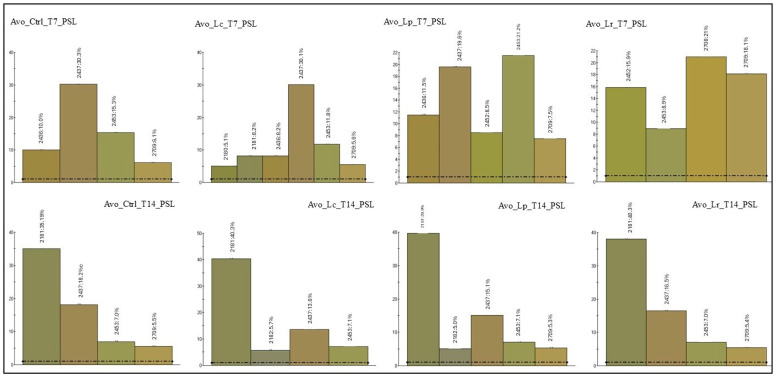
Colour spectrum primary shelf life. The graph shows the colour components that exceed a 5% representation threshold to identify the predominant hues in each sample. Dotted lines mark a value of 1%, representing the lowest threshold detectable by the instrument.

**Figure 5:**
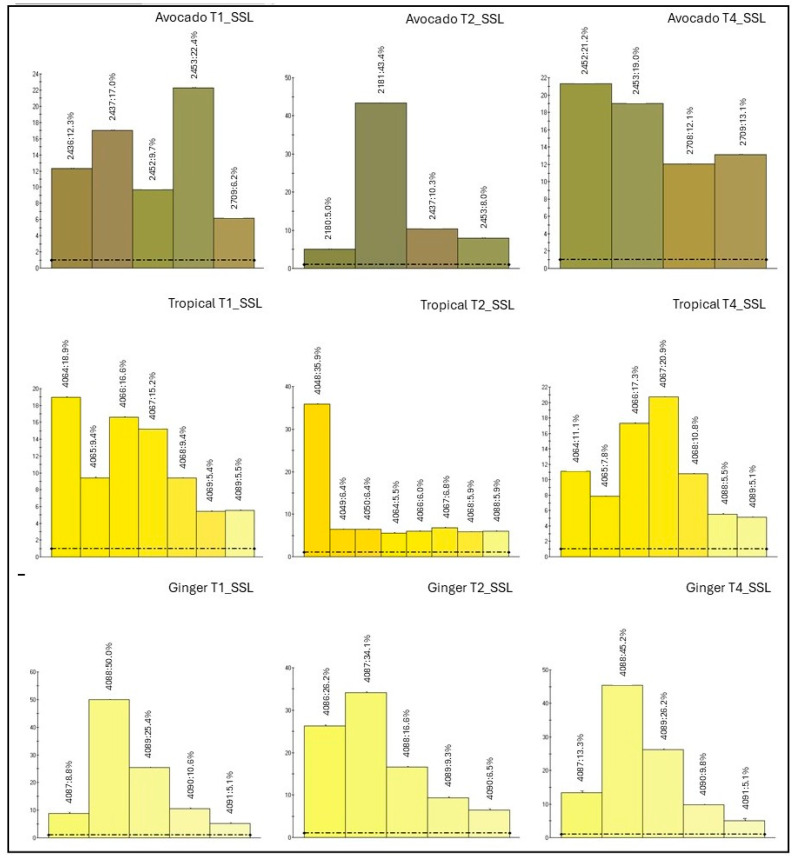
Colour spectrum secondary shelf life. The graph shows the colour components that exceed a 5% representation threshold to identify the predominant hues in each sample. Dotted lines mark a value of 1%, representing the lowest threshold detectable by the instrument.

For the primary shelf life assessment, images were collected at time points T7 and T14. These images were analysed using a colour spectrum approach, focusing only on colour components that exceeded a 5% representation threshold to identify the predominant hues in each sample. The chromatic analysis at T7 and T14 revealed notable differences in how the probiotic strains affected colour stability, depending on both the type of probiotic and the extract matrix. In the avocado extract (Figure 4), inoculation with *L. reuteri* at T7 caused a marked change in the colour profile, as indicated by the appearance of new colours (e.g., colour code 2708) and a distribution pattern distinct from the control. However, this effect diminished over time: by T14, the colour profile of the inoculated samples closely resembled that of the control, suggesting colour stabilisation. This may be due to microbial adaptation, system saturation, or inherent colour changes in the juice itself, some of which were also seen in the non-inoculated control. The strains *L. casei* and *L. plantarum* had a milder effect on the colour profile as early as T7, with values similar to the control and consistent through T14. This suggests a lower interaction between these strains and the extract pigments. In the tropical extract, *L. casei* consistently had the least impact on colour, with a chromatic profile nearly identical to the control at both time points. In contrast, *L. reuteri* and *L. plantarum* caused more noticeable colour changes at T7, with *L. plantarum* especially shifting the profile toward lighter tones. By T14, these changes were less evident, although *L. reuteri*-inoculated samples still showed an increased presence of colour code 4048, indicating a possible specific effect on certain pigments. In the ginger extract, the colour composition remained stable over time and was minimally affected by probiotic inoculation. All probiotic strains, including *L. reuteri*, yielded colour values comparable to the control at both T7 and T14. These results suggest that ginger extract has a higher resistance to microorganism-induced colour changes, likely due to its chemically stable composition or the presence of antioxidant compounds. For the secondary shelf life assessment (Figure 5), colour spectrum analysis revealed a slight shift at T2, especially in avocado and tropical extracts, where new colour codes appeared compared to T1. However, by T4, the colour tones had mostly reverted to their original state. Once again, ginger extract showed the greatest chromatic stability across the entire observation period. In the study conducted by de Oliveira et al., the effect of *Lactobacillus rhamnosus*, *Lactobacillus plantarum*, and *Lactobacillus acidophilus* in mixed mango and carrot juices was evaluated. The results showed that the addition of probiotics did not cause significant changes in the colorimetric parameters L*, a*, and b* during the storage period, indicating that the probiotics did not perceptibly affect the juice colour from a consumer standpoint [46]. The use of an electronic eye in this study underscores their remarkable precision and consistency in capturing subtle yet meaningful chromatic variations that might elude human perception. These tools offer a standardised, objective approach to assessing sensory attributes such as colour, an essential quality marker in consumer acceptance [47].

Moreover, their application in shelf life evaluation provides valuable insights into how microbial or chemical changes influence the organoleptic properties of food matrices over time. Such technologies not only enhance the reliability of product development and quality control processes but also support the formulation of more stable and appealing functional beverages [48].

### 3.5. Volatile Profile

The volatile fraction of the fruit and fruit/vegetable extracts was particularly rich and complex. A total of 165 volatile organic compounds (VOCs) belonging to various chemical classes were detected, including 12 aldehydes, 9 ketones, 22 alcohols, 4 acids, 37 esters, and 78 terpenoids. Additionally, two furanoic compounds and one nitrile derivative were also identified. The contribution of each chemical group to the total volatile profile varied among the investigated fruit and fruit–vegetable extracts, as well as depending on storage time and probiotic strain.

**Figure 6:**
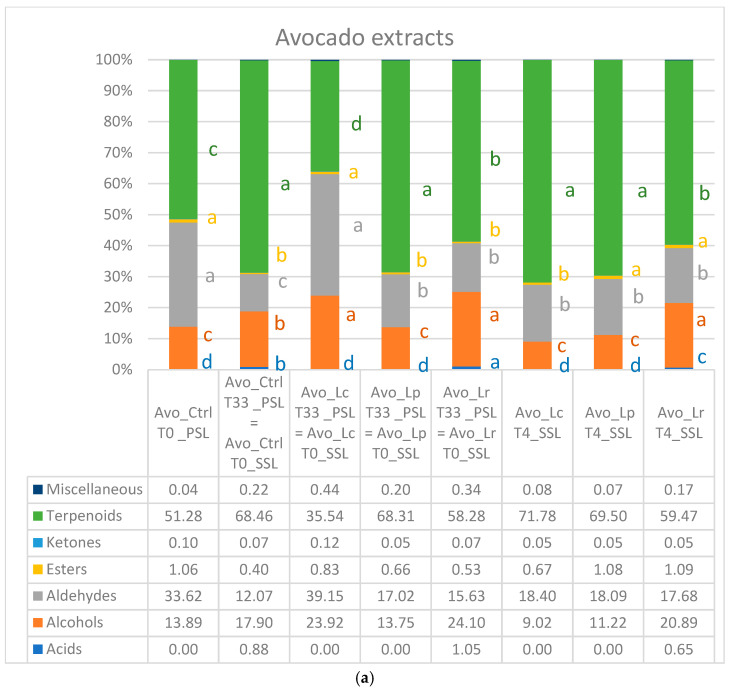

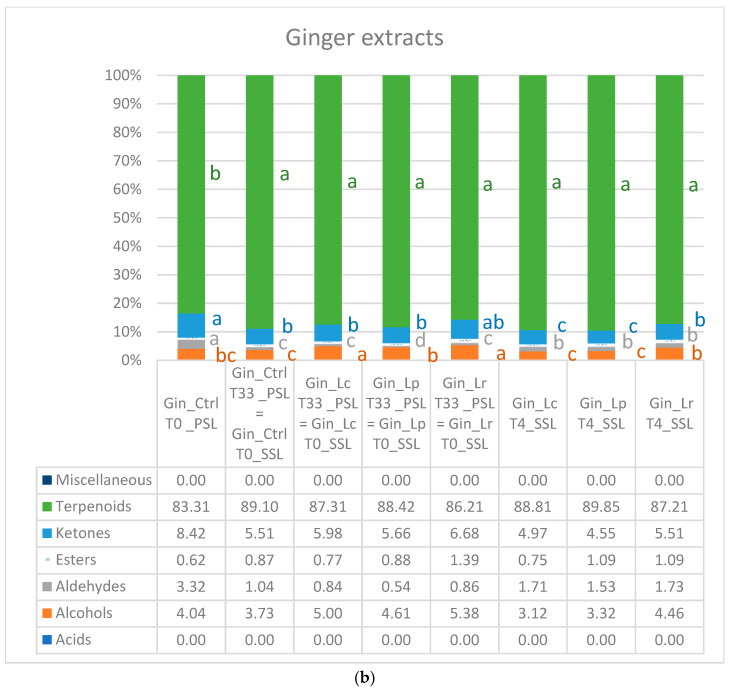

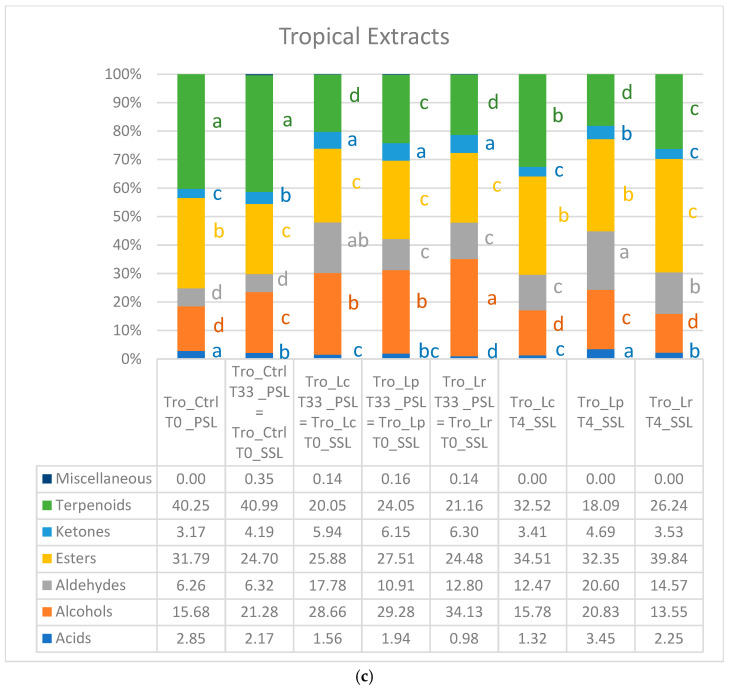
Volatile fraction composition (%) by classes of substances in (**a**) avocado, (**b**) ginger, and (**c**) tropical extracts during primary and secondary shelf life under refrigerated storage conditions. Different letters indicate statistically significant differences among samples by Turkey’s multiple range test at *p* < 0.05.

In avocado extracts, the volatile fraction primarily consisted of terpenoids, aldehydes, and alcohols, with ketones and esters present in very low percentages (Figure 6a). Limonene was by far the most abundant VOC, ranging from 26.32% to 54.51%. It was followed in decreasing order by hexanal (10.33% to 29.12%), hexanol (7.28% to 15.45%), and (E)-2-hexenal (1.21% to 6.32%). Other notable compounds included the hydrocarbon monoterpenes and sesquiterpenes such as β-pinene, (E)-α-bergamotene, myrcene, α-pinene, α-cubebene, and α-ylangene (Table S1).

The volatile fraction of ginger extracts was constituted almost entirely by terpenoids (Figure 6b), which accounted for more than 83% of the entire volatile fraction. Ketones, alcohols, and aldehydes were below 9%, whereas acids were not represented. The main VOCs were the monoterpenes eucalyptol (7.81–20.02%), neral (3.67–10.93%), β-phellandrene (1.15–12.48%), and geranial (2.71–12.49%). Furthermore, 2-undecanone (2.06–5.15%) and 2-nonanone (1.81–3.14%) prevailed among ketones (Table S2).

The tropical extracts exhibited a more complex volatile composition, with terpenoids, esters, and alcohols well represented, and ketones and acids present to a lesser extent (Figure 6c). In addition to limonene (7.49% to 26.37%) and 3-carene (4.93% to 11.22%), which are the typical VOCs of mango fruits, esters were the most prevalent compounds. The predominant esters included 2-methylbuthyl acetate (2.91–9.96%), 2-pentyl acetate (5.02–8.68%), and 2-penthyl butanoate (1.75–3.78%), which are the VOCs responsible for apple and banana aroma and flavour notes. Also, C6-aldehydes and alcohols were well represented, with hexanal ranging from 2.08% to 6.45% and hexanol from 6.01% to 12.19% (Table S3).

The composition of the volatile profiles of the examined extracts aligns with their formulations. It is known that esters, terpenoids, and alcohols are the primary VOCs in fruit juices [49], as seen in the case of the tropical extract, which is formulated entirely from a blend of mixed fruits. Conversely, in ginger extracts, the predominance of terpenoids has to be ascribed to the spice itself, as terpenes make up a significant portion of the ginger volatile fraction [50]. Key ginger VOCs include β-phellandrene, α-zingiberene, α-farnesene, neral, geranial, eucalyptol, and camphene [51], which were also among the most represented aroma compounds in our ginger extracts. Similarly, the high levels of terpenes, particularly limonene, along with the so-called green leaf volatiles (such as hexanal, hexanol, and (E)-2-hexenal) found in avocado extracts, correspond to the inclusion of avocado fruit [52] and spinach [53] in their formulation.

During refrigeration storage, both qualitative and quantitative changes occurred in the composition of the volatile fraction of the fruit and fruit–vegetable extracts. Specifically, in the avocado extracts, we observed an increase in 1-butanol and 2-methylbutanol during the PSL, as well as an increase in γ-terpinene and caryophyllene percentages throughout the entire storage time (both PSL and SSL). Additionally, eucalyptol appeared during SSL. In the ginger extracts, the primary changes occurred in the terpenoid groups, with a slight increase in the levels of oxygenated monoterpenes, especially terpinene-4-ol, throughout the entire storage period, both before and after the package was opened. Finally, in the tropical extracts, the percentages of ethanol, 2-pentyl acetate, and 2-hexenal increased, while new compounds, such as 2-methyl-1-butanol, emerged during the storage period. However, a decrease in the percentages of ethanol and 2-methyl-1-butanol was observed after the package was opened, likely due to their high volatility. The changes observed in the terpenoid compounds of the control and probiotic fruit and fruit–vegetable extracts during storage can primarily be attributed to acid-catalysed reactions. Fruit extracts are aqueous solutions with low pH levels that can promote rearrangement and isomerization reactions, as well as hydrolysis and oxidation processes. So, terpinene can arise primarily through limonene or α-pinene rearrangement, whereas oxygenated derivatives—especially terpinen-4-ol—result from oxidation [54]. Additionally, terpenoid compounds may result from the hydrolysis of their glycosides due to enzymes released by microorganisms or as secondary metabolites from the microorganisms themselves.

**Figure 7:**
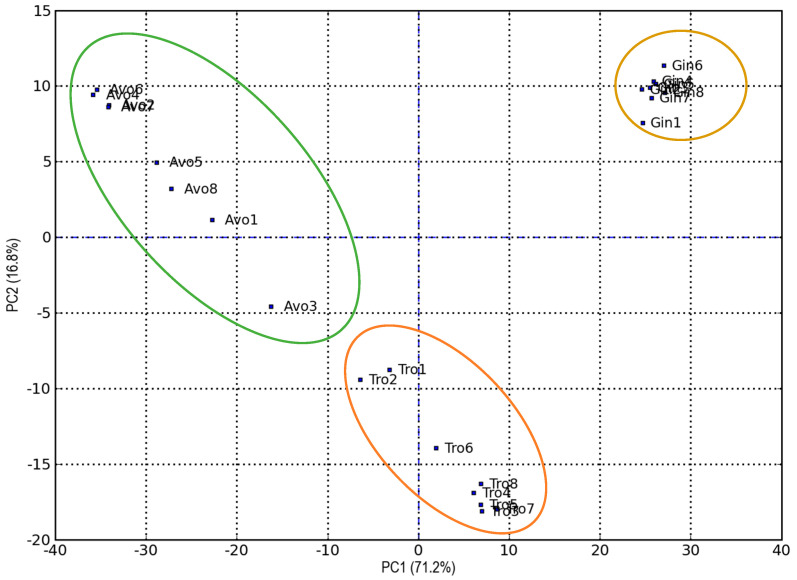
PCA score plot of volatile aroma compounds of fruit and fruit–vegetable extracts during primary and secondary shelf life under refrigerated storage conditions. 1 = Ctrl T0_PSL; 2 = Ctrl T33_PSL; 3 = Lc T33_PSL = Lc T0_SSL; 4 = Lp T33_PSL = Lp T0_SSL; 5 = Lr T33_PSL = Lr T0_SSL; 6 = Lc T4_SSL; 7 = Lp T4_SSL; 8 = Lr T4_SSL. The green circle groups the avocado extract samples; the orange circle includes the tropical extract samples, and the yellowish circle includes the ginger extract samples.

To gain a clearer understanding of the differences in the volatile aroma profiles of the examined extracts according to storage time and strains, PCA (Principal Component Analysis) was performed. Figure 7 displays the PCA score plot for the first two principal components (PCs), which together account for 88% of the total variance, with 71.2% explained by PC1 and 16.8% by PC2.

The figure highlights a clear separation of the samples based on the type of extract. Ginger extracts are located in the first quadrant (PC1 and PC2 both positive), very far from the others. Avocado extracts are found between the second (PC1 negative, PC2 positive) and third (PC1 and PC2 both negative) quadrants, whereas tropical extracts are positioned between the third and the fourth (PC1 positive, PC2 negative) quadrants. Within the group of ginger extracts, the different samples appear closely clustered, indicating only slight variations in the volatile fraction composition based on storage time and strain. Examining the group of tropical extracts, we observe that they are further classified by strain. Probiotic extracts are found in the fourth quadrant, while the control extracts are positioned in the third quadrant, regardless of the storage period. Avocado extracts show the highest variability. Within this group, the samples are further differentiated based on storage time and strain. The extracts fortified with *L. reuteri* are close to the fresh control sample (Avo_Ctrl T0_PSL), while the remaining samples are close to Avo_Ctrl T33 PSL. Avo_Lc T33_PSL stands alone in the third quadrant. The VOCs that predominantly contribute to sample separation in the PCA are listed in Table 6.

### 3.6. Sensory Stability

Hedonic tests were conducted to assess consumer acceptability of the developed probiotic fruit and fruit–vegetable extracts. The consumer panel evaluated both the control and probiotic-enhanced extracts by rating their appearance, odour, flavour, taste, and aftertaste. This assessment occurred 33 days after probiotic inoculation, marking the transition from PSL to SSL (T33_PLS = T0_SSL).

The sensory attribute scores for the fruit and fruit–vegetable extracts ranged from 6.94 ± 0.14 to 8.94 ± 0.13 (Table 7), indicating that consumers highly appreciated these products. Additionally, variance analysis revealed no statistically significant differences in sensory scores based on the probiotic strain used. This suggests that the addition of the selected probiotics did not affect the sensory profile of the studied plant-based extracts.

The high acceptability scores observed in this study for the selected fruit and fruit–vegetable extracts can be attributed to the overall quality of these products. As stated in Section 2.1, these extracts consist of 100% fruits and vegetables, with no added water, sugar, flavourings, or colourants, and they have been minimally processed. Previous sensory studies have highlighted that a combination of high-pressure processing (HPP) and low-temperature storage preserves the properties of fresh fruit and vegetable juices, as well as smoothies, more effectively than thermal pasteurisation [55]. Fruit juices and smoothies that undergo HPP exhibit “fresh-like” sensory properties, particularly in terms of aroma, taste, and flavour. They lack the artificial fruit aroma and flavour, cooked aroma and flavour, oxidised flavour, and off-flavour, commonly found and perceived in thermal-treated juices and smoothies. As a result, they are more readily accepted by consumers. Therefore, the fruit and fruit–vegetable extracts examined in this study meet consumer demand for high-quality, additive-free fruit and vegetable products with “fresh-like” sensory properties, leading to their high acceptability. Moreover, the utilisation of these extracts for delivering viable probiotic bacteria without fermenting them (i.e., as probiotic non-fermented beverages) allowed for the preservation of their valued sensory characteristics. Although some changes in pH, acidity, and volatile aroma profile were observed during the cold storage of the fruit and fruit/vegetable extracts—likely due to the metabolic activity of probiotic bacteria—their slight extent ensured no noticeable sensory differences between probiotic and non-probiotic extracts.

Previous research has shown that the consumer acceptability of probiotic fruit juices can change over time during storage. de Oliveira et al. [46] found that adding different strains of lactic acid bacteria (e.g., *Lactobacillus rhamnosus*, *Lactobacillus plantarum*, and *Lactobacillus acidophilus*) to mango and carrot mixed juices did not affect their acceptance at the time of production. However, after 35 days of storage at 8 °C, the acceptability of these juices decreased. The same authors reported a significant increase in titratable acidity and a decrease in pH of the probiotic juices during storage, attributing a negative impact to these changes on the juice sensory quality. Kardooni et al. [43] demonstrated that the storage temperature significantly impacted the consumer acceptability of probiotic orange juices containing *Lactobacillus acidophilus*. Juices stored at refrigeration temperature (4 °C) received higher acceptance scores compared to those stored at 21 °C. Their findings reinforce the negative correlation between increased acidity and consumer preference in fruit juices. Higher storage temperatures accelerate the metabolic activity of Lactobacilli, which ferment citrate and produce diacetyl, ultimately contributing to the undesirable “butter” off-flavour characteristic of spoilage juices [56]. In our study, the juice production processes and storage conditions minimised bacterial metabolic activity, both probiotic and spoilage-related, as demonstrated by pH stability and only minor variations in acidity. This ensures high sensory stability and strong consumer acceptance of both control and probiotic extracts throughout the storage period.

To fully assess the sensory stability of the developed probiotic extracts, triangle tests were conducted after package opening (SSL). This involved comparing the control and probiotic extracts that were just opened (T0_SSL) with their counterparts that had been opened for 1 day (T1_SSL) or 4 days (T4_SSL). The results of the triangle test, involving thirty-six judges, are presented in Table 8.

The data indicated that the percentage of assessors able to identify and describe sensory differences between the extracts at various storage times during SSL remained below 34.4%, except for the Avo_Lc T4_SSL sample. In this particular case, 67.3% of the assessors were able to distinguish it from the freshly opened Avo_Lc T0_SSL sample. According to Lawless & Heymann [57], a minimum of 24 correct responses is required to demonstrate a significant difference between samples. Consequently, we can conclude that there were no significant sensory differences (*p* > 0.05) between the extract samples at different storage times during SSL, except for the Avo_Lc T4_SSL sample. In this instance, assessors perceived the Avo_Lc T4_SSL sample as less acidic and sweeter than the Avo_Lc T0_SSL sample, expressing a preference for it. These findings were consistent with the variations in total acidity (TA) observed in the avocado extracts during their secondary shelf life. After the package was opened, the TA of the probiotic avocado juices decreased over time. This reduction in acidity likely enhanced the perception of sweetness by shifting the balance between acidity and sweetness, contributing to a more pleasant sensory perception. Based on previous research, sweetness consistently emerges as the primary factor influencing consumer preference across a wide range of foods and beverages. This effect is particularly pronounced in products where sweetness is not only a characteristic sensory attribute but also an anticipated and essential element of the overall flavour experience, such as biscuits, desserts, snacks, fruit juices, and soft drinks [58,59].

## 4. Conclusions

The results indicate that the three fruit and fruit–vegetable extracts serve as promising matrices for the delivery of free probiotic cells, successfully maintaining the viability of the selected strains, *L. reuteri*, *L. casei*, and *L. plantarum*, above the minimum recommended threshold of 6 log CFU/mL throughout both primary and secondary shelf life refrigerated storage periods. The study also enabled the verification of microbial viability throughout their entire shelf life without the influence of protective technologies, serving as a foundational step for future formulation strategies.

The nutritional and phytochemical properties of the beverages, including polyphenol, flavonoid, fibre, and lipid content, reflect the composition of their plant-based ingredients and are influenced by the nature of the matrix itself.

From a microbiological perspective, the products consistently met the hygienic standards, with no detectable contamination or significant alteration during storage. Physicochemical stability, particularly in terms of pH and titratable acidity, remained within acceptable ranges across most strain–extract combinations, with minor fluctuations attributable to probiotic metabolic activity or natural chemical changes within the matrix. Additionally, colour and aromatic profiles were generally well preserved, with only minimal changes—most notably in the avocado-based extracts—linked to acidity-driven reactions and the volatility of certain compounds. However, these variations did not significantly impact the sensory profile perceived by the consumer panel, except for the avocado extract fortified with *L. casei* at the end of the SSL. Sensory analysis confirmed a high degree of consumer acceptability across all formulations, along with good sensory stability during both PSL and SSL.

Compared to encapsulated, freeze-dried, or spray-dried probiotics, the use of free cells avoids stressors such as dehydration, elevated temperatures, or exposure to encapsulating agents, factors that may compromise cell functionality or delay metabolic activity. This approach was especially suited for our experimental context focused on fresh, non-thermally treated beverages. Nonetheless, we acknowledge that encapsulation offers clear advantages for shelf life extension and controlled delivery, and future studies will consider microencapsulation as a comparative strategy.

## Figures and Tables

**Table 1:** Experimental design.

Formulations of Fruit and Fruit–Vegetable Extracts					
	No strains	*L. casei*	*L. plantarum* LP09	*L. reuteri* DSM 17938	
Avocado extract	Avo_Ctr	Avo_Lc	Avo_Lp	Avo_Lr	
Ginger extracts	Gin_Ctrl	Gin_Lc	Gin_Lp	Gin_Lr	
Tropical extract	Tro_Ctrl	Tro_Lc	Tro_Lp	Tro_Lr	
Sampling time/days					
Primary Shelf life	0	7	14	21	33
	T0_PSL	T7_PSL	T14_PSL	T21_PSL	T33_PSL
Secondary Shelf life	0	1	2	4	
	T0_SSL	T1_SSL	T2_SSL	T4_SSL	

**Table 2:** Proximate composition and nutritional value of fruit and fruit/vegetable extract control samples.

	Avocado Extract Avo_Ctrl	Ginger Extract Gin_Ctrl	Tropical Extract Tro_Ctrl
Moisture	84.78 ± 0.83	86.61 ± 0.84	87.57 ± 0.81
Fat	3.11 ± 0.01 a	0.49 ± 0.00 b	0.51 ± 0.00 b
of which saturated fat	0.80 ± 0.00 a	0.09 ± 0.00 b	0.09 ± 0.00 b
Protein	0.81 ± 0.00 a	0.52 ± 0.00 b	0.69 ± 0.00 b
Carbohydrates	11.00 ± 0.04 b	12.25 ± 0.03 a	11.02 ± 0.05 b
of which sugar	9.41 ± 0.04	9.08 ± 0.04	10.09 ± 0.04
Fibre	1.59 ± 0.00 b	3.15 ± 0.01 a	0.42 ± 0.00 c
Ash	0.30 ± 0.00 a	0.15 ± 0.00 c	0.21 ± 0.00 b
of which NaCl	0.02 ± 0.00 a	0.01 ± 0.00 b	0.01 ± 0.00 b
Energy/Kcal	75	46	57

Data are expressed as mean ± SE of six replicates. Different letters in same row indicate significant differences at *p* < 0.05 among samples by Tukey’s multiple range test.

**Table 3:** Phytochemical content in fruit and fruit/vegetable extract control samples.

	Avocado Extract Avo_Ctrl	Ginger Extract Gin_Ctrl	Tropical Extract Tro_Ctrl
TPC (mg AGE/100 mL)	44.1 ± 1.2 a	27.5 ±0.6 b	45.0 ± 1.1 a
TFC (mg catechin/mL)	26.7 ± 0.2 c	37.1 ± 0.9 b	49.3 ± 0.2 a

Data are expressed as mean ± SE of six replicates. Different letters in same row indicate significant differences at *p* < 0.05 among samples by Turkey’s multiple range test.

**Table 4:** Variations in pH values during primary and secondary shelf life under refrigerated storage conditions.

Primary Shelf Life					
Avocado Extracts		Ginger Extracts		Tropical Extracts	
Strain	pH	Strain	pH	Strain	pH
Avo_Ctrl	4.09 ± 0.03 a	Gin_Ctrl	4.01 ± 0.04 a	Tro_Ctrl	3.79 ± 0.04 a
Avo_Lc	4.13 ± 0.03 a	Gin_Lc	4.04 ± 0.04 a	Tro_Lc	3.76 ± 0.04 a
Avo_Lp	4.10 ± 0.03 a	Gin_Lp	4.05 ± 0.04 a	Tro_Lp	3.74 ± 0.04 a
Avo_Lr	4.13 ± 0.03 a	Gin_Lr	4.02 ± 0.04 a	Tro_Lr	3.75 ± 0.04 a
Storage time	pH	Storage time	pH	Storage time	pH
T0_PSL	4.01 ± 0.03 c	T0_PSL	3.98 ± 0.04 a	T0_PSL	3.74 ± 0.04 a
T7_PSL	4.13 ± 0.03 b	T7_PSL	3.99 ± 0.04 a	T7_PSL	3.67 ± 0.04 a
T14_PSL	4.10 ± 0.03 b	T14_PSL	4.02 ± 0.04 a	T14_PSL	3.83 ± 0.04 a
T21_PSL	4.18 ± 0.03 a	T21_PSL	4.11 ± 0.04 a	T21_PSL	3.80 ± 0.04 a
T33_PSL	4.15 ± 0.03 b	T33_PSL	4.06 ± 0.04 a	T33_PSL	3.76 ± 0.04 a
Significance					
Strain	ns		ns		ns
Storage Time	**		ns		ns
Strain × Storage time	ns		ns		ns
**Secondary Shelf Life**					
**Avocado extracts**		**Ginger extracts**		**Tropical extracts**	
Strain	pH	Strain	pH	Strain	pH
Avo_Ctrl	3.86 ± 0.07 a	Gin_Ctrl	3.82 ± 0.06 a	Tro_Ctrl	3.63 ± 0.06 a
Avo_Lc	4.04 ± 0.07 a	Gin_Lc	3.93 ± 0.06 a	Tro_Lc	3.56 ± 0.06 a
Avo_Lp	3.98 ± 0.07 a	Gin_Lp	3.88 ± 0.06 a	Tro_Lp	3.63 ± 0.06 a
Avo_Lr	3.92 ± 0.07 a	Gin_Lr	3.88 ± 0.06 a	Tro_Lr	3.64 ± 0.06 a
Storage time	pH	Storage time	pH	Storage time	pH
T0_SSL	4.14 ± 0.07 a	T0_SSL	4.06 ± 0.06 a	T0_SSL	3.77 ± 0.06 a
T1_SSL	3.89 ± 0.07 b	T1_SSL	3.83 ± 0.06 b	T1_SSL	3.63 ± 0.06 b
T2_SSL	3.89 ± 0.07 b	T2_SSL	3.77 ± 0.06 c	T2_SSL	3.48 ± 0.06 c
T4_SSL	3.87 ± 0.07 b	T4_SSL	3.85 ± 0.06 b	T4_SSL	3.60 ± 0.06 b
Significance					
Strain	ns		ns		ns
Storage Time	*		*		*
Strain × Storage time	ns		ns		ns

Data are expressed as mean ± standard error of six replicates. Asterisks indicate significant effects of strain, storage time, and their interaction according to two-way ANOVA (* *p* < 0.05; ** *p* < 0.01; ns = not significant). Different letters in same column indicate significant differences by Turkey’s multiple range test at *p* < 0.05.

**Table 5:** Variation in titratable acidity (g citric acid/100 mL extract) during primary and secondary shelf life under refrigerated storage conditions.

		Primary Shelf Life			
Avocado Extracts		Ginger Extracts		Tropical Extracts	
Strain	TA (% CA)	Strain	TA (% CA)	Strain	TA (% CA)
Avo_Ctrl	0.394 ± 0.005 a	Gin_Ctrl	0.349 ± 0.005 a	Tro_Ctrl	0.644 ± 0.005 a
Avo_Lc	0.401 ± 0.005 a	Gin_Lc	0.344 ± 0.005 a	Tro_Lc	0.644 ± 0.005 a
Avo_Lp	0.399 ± 0.005 a	Gin_Lp	0.348 ± 0.005 a	Tro_Lp	0.638 ± 0.005 a
Avo_Lr	0.408 ± 0.005 a	Gin_Lr	0.348 ± 0.005 a	Tro_Lr	0.651 ± 0.005 a
Storage time	TA (% CA)	Storage time	TA (% CA)	Storage time	TA (% CA)
T0_PSL	0.408 ± 0.006 a	T0_PSL	0.355 ± 0.006 a	T0_PSL	0.622 ± 0.006 b
T7_PSL	0.364 ± 0.006 b	T7_PSL	0.345 ± 0.006 a	T7_PSL	0.651 ± 0.006 a
T14_PSL	0.406 ± 0.006 a	T14_PSL	0.344 ± 0.006 a	T14_PSL	0.646 ± 0.006 a
T21_PSL	0.412 ± 0.006 a	T21_PSL	0.346 ± 0.006 a	T21_PSL	0.650 ± 0.006 a
T33_PSL	0.412 ± 0.006 a	T33_PSL	0.347 ± 0.006 a	T33_PSL	0.651 ± 0.006 a
Significance					
Strain	ns		ns		ns
Storage Time	***		ns		**
Strain × Storage time	ns		ns		ns
		**Secondary Shelf Life**			
**Avocado extracts**		**Ginger extracts**		**Tropical extracts**	
Strain	TA (% CA)	Strain	TA (% CA)	Strain	TA (% CA)
Avo_Ctrl	0.442 ± 0.004 a	Gin_Ctrl	0.350 ± 0.004 a	Tro_Ctrl	0.678 ± 0.006 a
Avo_Lc	0.363 ± 0.004 b	Gin_Lc	0.343 ± 0.004 a	Tro_Lc	0.648 ± 0.006 b
Avo_Lp	0.351 ± 0.004 bc	Gin_Lp	0.343 ± 0.004 a	Tro_Lp	0.624 ± 0.006 c
Avo_Lr	0.346 ± 0.004 c	Gin_Lr	0.345 ± 0.004 a	Tro_Lr	0.627 ± 0.006 bc
Storage time	TA (% CA)	Storage time	TA (% CA)	Storage time	TA (% CA)
T0_SSL	0.412 ± 0.004 a	T0_SSL	0.347 ± 0.004 ab	T0_SSL	0.651 ± 0.006 a
T1_SSL	0.374 ± 0.004 b	T1_SSL	0.351 ± 0.004 a	T1_SSL	0.641 ± 0.006 a
T2_SSL	0.385 ± 0.004 b	T2_SSL	0.334 ± 0.004 b	T2_SSL	0.635 ± 0.006 a
T4_SSL	0.332 ± 0.004 c	T4_SSL	0.349 ± 0.004 ab	T4_SSL	0.650 ± 0.006 a
Significance					
Strain	***		ns		***
Storage Time	***		*		ns
Strain × Storage time	***		ns		ns

Data are expressed as mean ± standard error of six replicates. Asterisks indicate significant effects of strain, storage time, and their interaction according to two-way ANOVA (*** *p* < 0.001; ** *p* < 0.01; * *p* < 0.05; ns = not significant). Different letters in same column indicate significant differences by Turkey’s multiple range test at *p* < 0.05.

**Table 6:** Loading values of variables mostly influencing PC1 and PC2.

Variable	PC1	PC2	Variable	PC1	PC2
I Quadrant			III Quadrant		
Eucalyptol	0.230	0.376	(E)-2-Hexenal	−0.038	−0.258
a-Funebrene	0.182	0.292	γ-Terpinene	−0.087	0.087
ß-Phellandrene	0.124	0.203	Hexanol	−0.186	−0.253
Neral	0.117	0.185	Hexanal	−0.262	−0.008
Geranial	0.091	0.144	IV Quadrant		
Zingiberene	0.082	0.129	2-Methylbutyl acetate	0.02	−0.193
ß-Bisabolene	0.074	0.115	2-Pentyl acetate	0.019	−0.289
II Quadrant			3-Carene	0.011	−0.319
Limonene	−0.852	0.329	3-Methylbutyl butanoate	0.009	−0.159

**Table 7:** Sensory acceptability of control and probiotic fruit and fruit–vegetable extracts.

Avocado Extracts				
	Ctr T33_PSL = Ctr T0_SSL	Lc T33_PSL = Lc T0_SSL	Lp T33_PSL = Lp T0_SSL	Lr T33_PSL = Lr T0_SSL
Appearance	7.88 ± 0.18	8.06 ± 0.19	8.13 ± 0.18	7.94 ± 0.17
Odour	6.94 ± 0.14	7.13 ± 0.18	7.13 ± 0.18	6.94 ± 0.23
Flavour	7.44 ± 0.13	7.06 ± 0.21	6.94 ± 0.19	7.31 ± 0.12
Taste	7.44 ± 0.13	7.06 ± 0.21	6.94 ± 0.19	7.19 ± 0.16
Aftertaste	7.94 ± 0.23	7.81 ± 0.21	7.63 ± 0.15	7.38 ± 0.15
Overall	7.53 ± 0.13	7.43 ± 0.13	7.35 ± 0.11	7.35 ± 0.12
**Ginger Extracts**				
	Ctr T33_PSL = Ctr T0_SSL	Lc T33_PSL = Lc T0_SSL	Lp T33_PSL = Lp T0_SSL	Lr T33_PSL = Lr T0_SSL
Appearance	7.91 ± 0.15	8.06 ± 0.12	8.03 ± 0.14	7.94 ± 0.15
Odour	8.74 ± 0.13	8.35 ± 0.12	8.53 ± 0.16	8.64 ± 0.15
Flavour	8.44 ± 0.12	8.26 ± 0.14	8.46 ± 0.13	8.31 ± 0.12
Taste	7.44 ± 0.12	7.36 ± 0.14	7.29 ± 0.13	7.29 ± 0.16
Aftertaste	7.54 ± 0.12	7.41 ± 0.12	7.33 ± 0.14	7.38 ± 0.15
Overall	8.01 ± 0.13	7.89 ± 0.13	7.92 ± 0.12	7.91 ± 0.13
**Tropical Extracts**				
	Ctr T33_PSL = Ctr T0_SSL	Lc T33_PSL = Lc T0_SSL	Lp T33_PSL = Lp T0_SSL	Lr T33_PSL = Lr T0_SSL
Appearance	8.88 ± 0.15	8.26 ± 0.19	8.53 ± 0.18	8.94 ± 0.17
Odour	8.94 ± 0.13	8.89 ± 0.16	8.97 ± 0.18	8.94 ± 0.13
Flavour	8.44 ± 0.12	8.76 ± 0.19	8.64 ± 0.19	8.41 ± 0.12
Taste	8.87 ± 0.13	8.89 ± 0.19	8.94 ± 0.19	8.91 ± 0.16
Aftertaste	8.94 ± 0.13	8.81 ± 0.19	8.63 ± 0.14	8.38 ± 0.15
Overall	8.81 ± 0.15	8.72 ± 0.13	8.74 ± 0.11	8.71 ± 0.12

**Table 8:** Results from triangle test comparing control and probiotic extracts at different storage times during secondary shelf life under refrigerated storage conditions.

Avocado Extracts						
	Lc T0_SSL vs. Lc T1_SSL	Lp T0_SSL vs. Lp T1_SSL	Lr T0_SSL vs. Lr T1_SSL	Lc T0_SSL vs. Lc T4_SSL	Lp T0_SSL vs. Lp T4_SSL	Lr T0_SSL vs. Lr T4_SSL
Probability of correct answers. p_c_	0.143	0.201	0.175	0.673	0.182	0.364
Guessing probability. p_g_	0.33	0.33	0.33	0.33	0.33	0.33
Power	0.045	0.041	0.043	0.632	0.039	0.061
*p* -value	0.941	0.806	0.931	0.039	0.925	0.527
alpha	0.05	0.05	0.05	0.05	0.05	0.05
**Ginger Extracts**						
	Lc T0_SSL vs. Lc T1_SSL	Lp T0_SSL vs. Lp T1_SSL	Lr T0_SSL vs. Lr T1_SSL	Lc T0_SSL vs. Lc T4_SSL	Lp T0_SSL vs. Lp T4_SSL	Lr T0_SSL vs. Lr T4_SSL
Probability of correct answers. p_c_	0.273	0.196	0.206	0.273	0.183	0.179
Guessing probability. p_g_	0.33	0.33	0.33	0.33	0.33	0.33
Power	0.039	0.043	0.061	0.039	0.048	0.061
*p* -value	0.766	0.931	0.727	0.766	0.983	0.827
alpha	0.05	0.05	0.05	0.05	0.05	0.05
**Tropical Extracts**						
	Lc T0_SSL vs. Lc T1_SSL	Lp T0_SSL vs. Lp T1_SSL	Lr T0_SSL vs. Lr T1_SSL	Lc T0_SSL vs. Lc T4_SSL	Lp T0_SSL vs. Lp T4_SSL	Lr T0_SSL vs. Lr T4_SSL
Probability of correct answers. p_c_	0.271	0.273	0.275	0.255	0.269	0.364
Guessing probability. p_g_	0.33	0.33	0.33	0.33	0.33	0.33
Power	0.039	0.043	0.045	0.051	0.046	0.061
*p* -value	0.766	0.798	0.769	0.756	0.796	0.527
alpha	0.05	0.05	0.05	0.05	0.05	0.05

## Data Availability

The original contributions presented in the study are included in the article, further inquiries can be directed to the corresponding author.

## Supplementary Materials

The following supporting information can be downloaded at: https://www.mdpi.com/article/10.3390/foods14122148/s1. Table S1: Volatile profile composition (%) of avocado extracts during primary and secondary shelf life under refrigerated storage conditions; Table S2: Volatile profile composition (%) of ginger extracts during primary and secondary shelf life under refrigerated storage conditions; Table S3: Volatile profile composition (%) of tropical extracts during primary and secondary shelf life under refrigerated storage conditions.
