# Supplementary material for: Developing Novel Plant-Based Probiotic Beverages: A Study on Viability and Physicochemical and Sensory Stability
Source: Foods. 2025 Jun 19;14(12):2148. doi: 10.3390/foods14122148 (PMC12191691; doi:10.3390/foods14122148)
Supplement: Supplementary file 1 [file foods-14-02148-s001.zip › foods-3652322-supplementary.pdf]

Table S1 - Volatile profile composition (%) of Avocado extracts during primary and secondary shelf life under refrigerated storage conditions.

Compounds	RT	LRI	Avo_Ctrl T0 _PSL	Avo_Ctrl T33 _PSL = Avo_Ctrl T0_SSL	Avo_Lc T33 _PSL = Avo_Lc T0_SSL	Avo_Lp T33 _PSL = Avo_Lp T0_SSL	Avo_Lr T33 _PSL = Avo_Lr T0_SSL	Avo_Lc T4_SSL	Avo_Lp T4_SSL	Avo_Lr T4_SSL
Butanal	6.18	887	0.19±0.01c	0.12±0.01c	0.69±0.04a	0.21±0.01b	0.27±0.02b	tr d	tr d	tr d
Ethyl Acetate	6.32	893	0.19±0.01ab	0.06±0.00b	0.25±0.01a	0.07±0.00b	0.13±0.01ab	0.09±0.01b	0.09±0.01b	0.17±0.01ab
Methanol	6.45	899	0.51±0.03b	0.72±0.04a	0.74±0.04a	0.41±0.02b	0.49±0.03b	0.29±0.02c	0.32±0.02c	0.29±0.02c
Ethanol	7.14	928	0.22±0.01b	0.37±0.02ab	0.56±0.03a	0.27±0.02b	0.54±0.03a	0.12±0.01c	0.16±0.01c	0.33±0.02ab
Pentanal	8.25	976	0.33±0.02b	0.19±0.01a	0.83±0.05a	0.27±0.02bc	0.39±0.02b	0.21±0.01c	0.18±0.01c	0.22±0.01c
Methyl butanoate	8.34	980	tr b	0.01±0.00ab	0.01±0.00b	0.02±0.00a	0.03±0.00a	0.01±0.00ab	0.01±0.00ab	0.01±0.00ab
Methyl 2-methylbutanoate	8.96	1004	tr b	0.01±0.00ab	0.04±0.00a	0.02±0.00ab	0.01±0.00ab	0.01±0.00ab	0.02±0.00ab	0.02±0.00ab
α-Pinene	9.36	1015	1.55±0.09a	0.98±0.06b	0.93±0.05b	0.72±0.04bc	0.76±0.04bc	1.19±0.07ab	1.18±0.07ab	0.91±0.05b
α-Thujene	9.49	1019	0.43±0.02a	0.28±0.02b	0.35±0.02ab	0.19±0.01c	0.21±0.01c	0.44±0.03a	0.31±0.02b	0.27±0.02b
Camphene	10.66	1052	tr c	0.03±0.00b	0.03±0.00b	0.03±0.00b	0.02±0.00b	0.10±0.01b	0.08±0.00a	0.02±0.00b
Butyl acetate	10.89	1059	tr c	0.01±0.00b	0.05±0.00a	0.01±0.00b	0.02±0.00b	tr c	tr c	tr c
Hexanal	11.33	1071	26.30±1.52a	10.33±0.60b	29.12±1.68a	14.16±0.82b	13.36±0.77b	12.94±0.75b	13.06±0.75b	10.93±0.63b
β-Pinene	11.87	1086	3.35±0.19a	1.82±0.10c	2.26±0.13b	1.67±0.10c	1.75±0.10c	1.94±0.11c	1.89±0.11c	1.55±0.09c
Sabinene	12.28	1098	0.68±0.04a	0.21±0.01b	0.29±0.02b	0.22±0.01b	0.19±0.01b	0.36±0.02b	0.38±0.02b	0.27±0.02b
Propyl butanoate	12.60	1105	tr b	0.01±0.00a	0.02±0.00a	0.02±0.00a	0.02±0.00a	tr b	tr b	tr b
(E)-2-Pentenal	13.05	1115	nd[4] c	nd c	nd c	nd c	nd c	0.04±0.00b	0.04±0.00b	0.07±0.00a
3-Carene	13.50	1124	0.25±0.01a	0.21±0.01a	0.18±0.01a	0.19±0.01a	0.21±0.01a	0.13±0.01a	0.20±0.01a	0.14±0.01a
Myrcene	13.72	1129	2.04±0.12a	2.01±0.12a	1.14±0.07a	1.80±0.10a	1.75±0.10a	1.02±0.06a	1.07±0.06a	1.77±0.10a
1-Butanol	14.15	1138	nd c	3.55±0.20a	4.18±0.24a	2.81±0.16b	5.66±0.33a	0.46±0.03c	0.43±0.03c	2.76±0.16b
Ethyl (E)-2-butenoate	14.36	1142	0.15±0.01a	tr b	tr b	tr b	tr b	tr b	tr b	tr b
α-Terpinene	14.39	1143	0.59±0.03a	0.19±0.01b	0.09±0.01c	tr d	0.220.01b	0.07±0.00c	0.08±0.00c	0.21±0.01b
Limonene	15.38	1164	35.90±2.07b	53.21±3.07a	26.23±1.51bc	54.51±3.15a	45.732.64ab	54.79±3.16a	52.93±3.06a	44.27±2.56ab
1-Methyl-5-(1-methylethenyl)-cyclohexene	15.54	1168	0.15±0.01a	tr b	tr b	tr b	tr b	tr b	tr b	tr b
β-Phellandrene	15.67	1170	0.67±0.04a	0.19±0.01b	0.19±0.01b	0.21±0.01b	tr a	0.30±0.02ab	0.27±0.02b	0.23±0.01b
2-Pentyl butanoate	15.97	1177	0.26±0.02a	tr b	tr b	tr b	tr b	tr b	tr b	tr b
Eucalyptol	16.09	1179	tr b	tr b	tr b	tr b	tr b	0.13±0.01a	0.08±0.00a	0.01±0.00a
Butyl butanoate	16.29	1184	0.12±0.01b	tr b	0.33±0.02a	0.27±0.02a	0.25±0.01a	0.35±0.02a	0.41±0.02a	0.31±0.02a

2-Methyl-1-butanol	16.50	1188	nd a	1.15±0.07b	0.08±0.00d	0.43±0.02c	2.27±0.13a	0.09±0.01d	0.41±0.02c	0.95±0.05bc
3-Methyl-1-butanol	16.59	1190	0.36±0.02a	tr b	tr b	tr b	tr b	tr b	tr b	tr b
(E)-2-Hexenal	16.79	1194	6.32±0.37ab	1.21±0.07cd	8.22±0.47a	2.19±0.13c	1.45±0.08cd	4.69±0.27b	4.37±0.25b	6.01±0.35ab
γ-Terpinene	17.35	1206	0.18±0.01c	5.89±0.34a	tr d	5.11±0.29a	4.50±0.26b	6.35±0.37a	6.40±0.37a	5.22±0.30a
β-Ocimene	17.62	1212	0.45±0.03a	0.11±0.01b	0.05±0.00c	0.05±0.00c	tr d	0.11±0.01b	0.11±0.01b	0.09±0.01b
1-Pentanol	18.18	1224	0.44±0.03b	0.34±0.02bc	0.56±0.03a	0.20±0.01c	0.55±0.03a	0.13±0.01cd	0.16±0.01cd	0.35±0.02bc
p-Cymene	18.57	1233	0.19±0.01c	0.26±0.01b	0.78±0.04a	0.91±0.05a	0.79±0.05a	0.29±0.02b	0.24±0.01b	0.29±0.02b
Hexyl acetate	18.79	1237	tr c	0.10±0.01b	tr a	0.09±0.01b	tr c	tr c	0.35±0.02a	0.32±0.02a
Terpinolene	19.03	1242	nd a	0.33±0.02b	0.36±0.02b	0.31±0.02b	0.27±0.02b	0.56±0.03a	0.33±0.02b	0.30±0.02b
Hexanenitrile	20.20	1268	0.04±0.00c	0.11±0.01ab	0.20±0.01a	0.16±0.01ab	0.15±0.01ab	0.08±0.00b	0.07±0.00b	0.09±0.01b
2,5-Octanedione	21.18	1289	0.05±0.00a	0.04±0.00a	0.04±0.00a	0.02±0.00a	0.03±0.00a	tr b	tr b	tr b
(Z)-2-Heptenal	21.35	1292	0.38±0.02a	0.10±0.01b	0.20±0.01ab	0.09±0.01b	0.11±0.01b	0.36±0.02a	0.32±0.02a	0.28±0.02a
6-Methyl-5-hepten-2-one	21.86	1303	0.05±0.00a	0.03±0.00a	0.08±0.00a	0.03±0.00a	0.04±0.00a	0.05±0.00a	0.05±0.00a	0.05±0.00a
Hexanol	22.57	1318	11.50±0.66bc	11.15±0.64bc	17.24±1.00a	9.34±0.54bc	14.14±0.82ab	7.28±0.42c	9.11±0.53bc	15.45±0.89ab
(E)-3-Hexen-1-ol	23.02	1327	tr b	0.02±0.00a	0.02±0.00a	0.01±0.00a	0.01±0.00a	0.01±0.00a	0.01±0.00a	0.03±0.00a
(Z)-3-Hexen-1-ol	23.93	1346	tr c	0.04±0.00a	0.01±0.00bc	0.02±0.00ab	0.03±0.00ab	0.04±0.00a	0.02±0.00ab	0.04±0.00a
Nonanal	24.28	1353	tr c	0.12±0.01a	0.05±0.00b	0.06±0.00b	0.02±0.00bc	0.09±0.01ab	0.05±0.00b	0.08±0.00ab
(Z)-2-Hexen-1-ol	24.85	1365	0.58±0.03a	0.57±0.03a	0.41±0.02a	0.20±0.01b	0.40±0.02a	0.41±0.02a	0.44±0.03a	0.63±0.04a
Hexyl butanoate	25.12	1370	0.35±0.02a	0.12±0.01b	0.12±0.01b	0.12±0.01b	0.05±0.00c	tr d	tr d	0.19±0.01b
4-Methylpentyl 2-methylbutanoate	25.58	1380	tr d	0.08±0.00b	tr d	0.04±0.00c	0.03±0.00c	0.20±0.01a	0.19±0.01a	0.08±0.00b
(E)-2-Octenal	26.00	1388	0.11±0.01b	tr c	tr c	tr c	tr c	0.07±0.00a	0.06±0.00a	0.06±0.00a
p-Cymenene	26.26	1394	tr c	tr c	tr c	tr c	tr c	0.09±0.01a	0.08±0.00a	0.03±0.00b
1-Octen-3-ol	26.65	1402	0.06±0.00a	0.03±0.00a	0.05±0.00a	0.02±0.00a	0.02±0.00a	0.03±0.00a	0.06±0.00a	0.04±0.00a
α-Cubebene	26.87	1407	0.93±0.05a	0.46±0.03b	0.39±0.02c	0.37±0.02c	0.38±0.02c	0.53±0.03b	0.48±0.03b	0.66±0.04b
Acetic acid	27.11	1413	tr b	0.88±0.05a	tr b	tr b	0.74±0.04a	tr b	tr b	0.65±0.04a
6-Methyl-5-hepten-2-ol	27.17	1414	0.10±0.01a	tr b	0.07±0.00a	0.05±0.00a	tr b	0.06±0.00a	0.07±0.00a	tr b
Furfural	27.66	1426	tr d	0.11±0.01b	0.24±0.01a	0.04±0.00c	0.19±0.01a	tr d	tr d	0.08±0.00b
α-Ylangene	27.99	1433	0.90±0.05a	0.09±0.01c	tr d	0.10±0.01c	0.02±0.00d	0.26±0.02b	0.27±0.02b	0.14±0.01c
2-Ethyl-1-hexanol	28.36	1442	0.12±0.01a	tr b	tr b	tr b	tr b	0.07±0.00a	tr b	tr b
α-Copaene	28.40	1443	tr d	0.31±0.02b	0.27±0.02b	0.25±0.01b	0.17±0.01c	0.33±0.02b	0.32±0.02b	0.45±0.03a
Benzaldehyde	30.19	1485	tr c	0.01±0.00b	0.03±0.00a	0.03±0.00a	0.03±0.00a	tr c	tr c	0.03±0.00a

β-Cubebene	30.39	1490	tr c	0.03±0.00b	0.07±0.00a	0.07±0.00a	0.09±0.01a	0.07±0.00a	0.08±0.00a	0.03±0.00b
Linalool	30.75	1498	0.10±0.01a	0.05±0.00a	0.08±0.00a	0.05±0.00a	0.08±0.00a	0.08±0.00a	0.07±0.00a	0.06±0.00a
1-Octanol	31.19	1509	tr b	tr b	tr b	tr b	tr b	0.04±0.00a	0.02±0.00a	0.02±0.00a
(E)-α-Bergamotene	32.35	1537	2.38±0.14a	0.52±0.03bc	0.63±0.04dc	0.49±0.03bc	0.30±0.02c	0.81±0.05b	0.84±0.05b	0.81±0.05b
Caryophyllene	32.89	1551	nd c	1.02±0.06ab	1.01±0.06ab	0.92±0.05ab	0.60±0.03b	1.31±0.08a	1.34±0.08a	1.42±0.08a
Terpinen-4-ol	33.14	1557	0.11±0.01b	0.18±0.01a	0.11±0.01b	0.10±0.01b	0.13±0.01b	0.06±0.00c	0.08±0.00c	0.13±0.01b
Citronellyl acetate	35.49	1617	0.11±0.01a	tr b	tr b	tr b	tr b	tr b	tr b	tr b
α-Humulene	35.80	1625	tr c	0.04±0.00b	0.07±0.00a	0.04±0.00b	0.07±0.00a	0.07±0.00a	0.08±0.00a	0.06±0.00ab
Neral	36.46	1642	tr d	tr b	tr b	tr b	tr b	0.05±0.00a	0.03±0.00a	tr b
Germacrene D	37.34	1666	tr c	0.04±0.00ab	0.03±0.00b	0.01±0.00b	0.02±0.00b	0.09±0.01a	tr c	0.01±0.00b
β-copaene	37.38	1667	tr b	tr b	tr b	tr b	tr b	tr b	0.09±0.01a	0.07±0.00a
β-Bisabolene	38.20	1689	tr a	tr a	tr a	tr a	tr a	0.12±0.01b	0.10±0.01b	0.03±0.00c
Geranial	38.43	1695	0.32±0.02a	tr d	tr d	tr d	tr d	tr a	tr a	tr a
(E,E)-α-Farnesene	38.88	1708	tr b	tr b	tr b	tr b	tr b	0.10±0.01a	0.09±0.01a	tr b
δ-Cadinene	39.27	1719	tr b	tr b	tr b	tr b	tr b	0.04±0.00a	0.03±0.00a	0.03±0.00a
Hexanoic acid	42.38	1808	tr b	tr b	tr b	tr b	0.31±0.02a	tr b	tr b	tr b

[1] Linear retention index on polar column calculated according to the Van Den Dool and Kratz equation; [2] Percentage of peak area in Total Ion Current (TIC) chromatograms; [3] traces = inferior to 0.005%. [4] not detected = inferior to LOD. Data are expressed as the mean of three replicates ± SE. Different letters in the same row indicate statistically significant differences by Turkey's multiple range test at $p < 0.05$ during the refrigerated storage time.

Table S2 - Volatile profile composition (%) of Ginger extracts during primary and secondary shelf life under refrigerated storage conditions.

Compounds	RT	LRI	Gin_Ctrl T0 _PSL	Gin_Ctrl T33 _PSL = Gin_Ctrl T0_SSL	Gin_Lc T33 _PSL = Gin_Lc T0_SSL	Gin_Lp T33 _PSL = Gin_Lp T0_SSL	Gin_Lr T33 _PSL = Gin_Lr T0_SSL	Gin_Lc T4_SSL	Gin_Lp T4_SSL	Gin_Lr T4_SSL
α-Pinene	9.36	1015	0.99±0.06c	1.89±0.11a	1.42±0.08ab	1.19±0.07b	1.22±0.07b	1.28±0.07b	1.26±0.07b	0.79±0.05c
α-Thujene	9.49	1019	tr a	tr a	tr a	tr a	tr a	tr a	tr a	tr a
Camphene	10.66	1052	2.72±0.16c	5.14±0.30a	4.02±0.23ab	3.81±0.22b	3.75±0.22b	4.19±0.24ab	2.89±0.17c	2.26±0.13c
Butyl acetate	10.89	1059	tr c	0.10±0.01b	0.20±0.01a	0.16±0.01ab	0.20±0.01a	0.26±0.02a	0.12±0.01b	0.21±0.01a
Hexanal	11.33	1071	1.46±0.08a	0.56±0.03b	0.25±0.01c	0.12±0.01d	0.21±0.01c	0.70±0.04b	0.46±0.03bc	0.54±0.03b
β-Pinene	11.87	1086	0.14±0.01b	0.27±0.02a	0.19±0.01b	0.17±0.01b	0.16±0.01b	0.23±0.01a	0.17±0.01b	0.11±0.01b
2-Methylbutyl acetate	12.54	1104	0.39±0.02b	0.26±0.01c	0.36±0.02b	0.30±0.02b	0.38±0.02b	0.77±0.04a	0.26±0.02c	0.45±0.03ab
3-Carene	13.50	1124	tr c	0.08±0.00a	0.05±0.00a	0.05±0.00a	0.03±0.00b	0.11±0.01b	0.02±0.00b	0.02±0.00b
β-Pinene	13.90	1133	1.94±0.11c	3.89±0.22a	3.15±0.18a	2.87±0.17b	2.79±0.16b	2.67±0.15b	2.47±0.14b	0.81±0.05d
Pseudolimonene (= p-Mentha-1(7),8-diene)	14.09	1137	tr b	0.07±0.00a	0.04±0.00a	0.04±0.00a	0.04±0.00a	0.06±0.00a	0.05±0.00a	0.01±0.00b
α-Terpinene	14.39	1143	0.01±0.00c	0.21±0.01a	0.21±0.01a	0.16±0.01ab	0.18±0.01a	0.10±0.01b	0.11±0.01b	0.03±0.00c
Pentyl acetate	14.51	1146	0.03±0.00a	tr b	tr b	tr b	tr b	tr b	tr b	tr b
2-Heptanone	15.13	1159	tr c	tr c	tr c	tr c	tr c	0.19±0.01a	0.10±0.01b	0.22±0.01a
Limonene	15.38	1164	2.67±0.15b	4.04±0.23a	3.43±0.20ab	3.30±0.19ab	3.00±0.17ab	2.88±0.17b	2.88±0.17b	1.80±0.10c
1-Methyl-5-(1-methylethenyl)-cyclohexene	15.54	1168	tr c	0.15±0.01a	0.07±0.00b	0.08±0.00b	0.11±0.01ab	tr c	tr c	tr c
β-Phellandrene	15.67	1170	7.35±0.42b	12.48±0.72a	10.11±0.58a	9.31±0.54ab	8.93±0.52ab	1.15±0.06c	7.80±0.45b	6.13±0.35b
Eucalyptol	16.09	1179	11.56±0.67b	12.86±0.74b	14.35±0.83b	14.07±0.81b	16.96±0.98b	20.02±1.22a	7.81±0.45c	11.40±0.66b
Butyl butanoate	16.29	1184	0.03±0.00a	0.03±0.00a	0.04±0.00a	tr b	0.04±0.00a	0.05±0.00a	0.03±0.00a	0.04±0.00a
2-Methyl-1-butanol	16.50	1188	0.08±0.00a	0.06±0.00b	0.09±0.01a	0.11±0.01a	0.08±0.00a	tr c	0.06±0.00b	0.12±0.01a
(E)-2-Hexenal	16.79	1194	1.21±0.07a	0.29±0.02b	0.43±0.02b	0.29±0.02b	0.42±0.02b	0.84±0.05a	0.92±0.05a	0.95±0.05a
Butyl 2-methylbutanoate	16.99	1198	tr b	0.07±0.00a	tr b	0.08±0.00a	tr b	0.06±0.00a	tr b	tr b
γ-Terpinene	17.35	1206	0.11±0.01b	0.21±0.01a	0.14±0.01b	0.14±0.01b	0.12±0.01b	0.13±0.01b	0.15±0.01b	0.11±0.01b
Butyl 3-methylbutanoate	17.81	1216	0.19±0.01a	tr b	tr b	tr b	tr b	tr b	tr b	tr b
6-Methyl-2-heptyl acetate	18.46	1230	tr c	0.04±0.00b	0.12±0.01a	0.13±0.01a	0.12±0.01a	0.08±0.00ab	0.13±0.01a	0.12±0.01a
p-Cymene	18.57	1233	0.28±0.02a	0.26±0.02a	0.20±0.01ab	0.20±0.01ab	0.18±0.01b	0.15±0.01b	0.18±0.01b	0.12±0.01c
Hexyl acetate	18.79	1237	0.12±0.01a	0.05±0.00b	0.06±0.00b	0.05±0.00b	0.06±0.00b	0.12±0.01a	0.10±0.01a	0.14±0.01a
Terpinolene	19.03	1242	0.60±0.03b	0.91±0.05a	0.71±0.04a	0.69±0.04ab	0.56±0.03a	0.59±0.03b	0.74±0.04a	0.47±0.03b
2-Octanone	19.53	1253	0.02±0.00a	0.02±0.00a	0.02±0.00a	0.03±0.00a	0.03±0.00a	0.02±0.00a	0.01±0.00a	0.03±0.00a

Octanal	19.67	1256	0.14±0.01b	0.10±0.01a	0.05±0.00b	0.03±0.00b	0.07±0.00b	0.10±0.01a	0.05±0.00b	0.09±0.01a
2-Heptanol	21.06	1286	1.19±0.07a	1.27±0.07b	1.61±0.09a	1.40±0.08ab	1.69±0.10a	1.17±0.07b	0.82±0.05c	1.27±0.07b
(Z)-2-Heptenal	21.35	1292	0.27±0.02a	tr c	tr c	tr c	tr c	tr c	tr c	tr c
6-Methyl-5-hepten-2-one	21.86	1303	0.50±0.03a	0.45±0.03a	0.58±0.03a	0.55±0.03a	0.65±0.04a	0.35±0.02a	0.32±0.02a	0.49±0.03a
Hexanol	22.57	1318	0.21±0.01b	0.22±0.01b	0.42±0.02a	0.41±0.02a	0.43±0.02a	0.21±0.01b	0.21±0.01d	0.34±0.02ab
2-Nonanone	24.18	1351	2.76±0.16b	2.54±0.15b	2.60±0.15b	2.59±0.15b	3.14±0.18a	2.35±0.14b	1.81±0.10c	2.50±0.14b
Rosefuran	24.60	1360	0.12±0.01a	0.13±0.01a	0.12±0.01a	0.12±0.01a	0.11±0.01a	0.08±0.00a	0.12±0.01a	0.15±0.01a
(Z)-2-Hexen-1-ol	24.85	1365	0.02±0.00a	0.02±0.00a	0.02±0.00a	0.01±0.00a	0.02±0.00a	tr b	0.01±0.00a	0.02±0.00a
Hexyl butanoate	25.12	1370	0.03±0.00a	tr b	tr b	tr b	tr b	tr b	tr b	tr b
Perillene	25.36	1375	0.12±0.01a	0.10±0.01a	0.06±0.00b	0.10±0.01a	0.05±0.00b	0.05±0.00b	0.10±0.01a	0.08±0.00ab
4-Methylpentyl 2-methylbutanoate	25.58	1380	0.06±0.00a	0.04±0.00a	0.05±0.00a	0.02±0.00a	0.04±0.00a	0.02±0.00a	0.04±0.00a	0.06±0.00a
(E)-2-Octenal	26.00	1388	0.25±0.01a	0.07±0.00c	0.06±0.00c	0.06±0.00c	0.09±0.00b	0.06±0.00c	0.10±0.01b	0.11±0.01b
p-Cymenene	26.26	1394	0.08±0.00a	0.04±0.00a	0.05±0.00a	0.05±0.00a	0.06±0.00a	0.05±0.00a	0.05±0.00a	0.05±0.00a
2-Nonyl acetate	27.14	1413	0.07±0.00a	0.03±0.00a	0.04±0.00a	0.03±0.00a	0.04±0.00a	0.03±0.00a	0.06±0.00a	0.07±0.00a
δ-Elemene	27.51	1422	tr c	0.04±0.00b	0.05±0.00b	0.06±0.00b	0.03±0.00b	0.06±0.00b	0.10±0.01a	0.04±0.00b
α-Ylangene	27.99	1433	0.16±0.01a	tr b	tr b	tr b	tr b	tr b	tr b	tr b
ε-Amorphene	28.08	1436	tr c	0.09±0.01b	0.09±0.01b	0.13±0.01b	0.08±0.00b	0.07±0.00b	0.23±0.01a	0.11±0.01b
2-Ethyl-1-hexanol	28.36	1442	0.34±0.02bc	0.34±0.02bc	0.49±0.03b	0.32v0.02bc	0.60±0.03a	0.25±0.01c	0.70±0.04a	0.38±0.02bc
α-Copaene	28.40	1443	0.29±0.02a	0.14±0.01b	tr d	0.25±0.01a	tr d	0.16±0.01b	0.09±0.01c	0.23±0.01a
Decanale	28.83	1453	tr c	0.02±0.00bc	0.06±0.00a	0.04±0.00b	0.07±0.00a	0.01±0.00bc	tr c	0.04±0.00b
Nonan-2-ol	29.62	1472	1.95±0.11b	1.64±0.09bc	2.13±0.12a	1.96±0.11b	2.30±0.13a	1.40±0.08c	1.31±0.08c	2.08±0.12a
2-Bornanone	29.71	1474	0.29±0.02a	0.13±0.01b	tr c	tr c	0.33±0.02a	0.34±0.02a	0.21±0.01a	tr c
Linalool	30.75	1498	3.30±0.19b	3.10±0.18b	3.47±0.20b	3.10±0.18b	4.19±0.24a	2.98±0.17a	2.16±0.12a	3.22±0.19b
1-Octanol	31.19	1509	0.18±0.01b	0.12±0.01b	0.15±0.01b	0.29±0.02a	0.17±0.01b	0.06±0.00c	0.20±0.01ab	0.14±0.01b
(Z)-p-Menth-2-en-1-ol	31.53	1517	0.49±0.03ab	0.36±0.02b	0.61±0.04a	0.53±0.03a	0.69±0.04a	0.31±0.02b	0.26±0.01b	0.51±0.03a
Isopulegol	31.94	1527	0.63±0.04a	0.26±0.02b	0.27±0.02b	0.26±0.01b	0.31±0.02b	0.25±0.01b	0.42±0.02a	0.46±0.03ab
Bornyl acetate	32.27	1536	0.67±0.04a	0.20±0.01c	0.32±0.02a	0.44±0.03b	0.35±0.02b	0.43±0.02b	0.40±0.02b	0.39±0.02b
Fenchyl alcohol	32.33	1537	tr b	0.10±0.01a	0.13±0.01a	tr b	0.13±0.01a	tr b	0.16±0.01b	0.12±0.01a
(E)-α-Bergamotene	32.35	1537	tr c	tr c	tr c	tr c	tr c	0.10±0.01b	0.09±0.01a	0.18±0.01a
β-Elemene	32.63	1545	0.57±0.03a	0.16±0.01c	0.32±0.02b	0.31±0.02b	0.17±0.01a	0.25±0.01bc	0.24±0.01b	tr d
2-Undecanone	33.05	1555	5.15±0.30a	2.49±0.14b	2.78±0.16b	2.50±0.14b	2.86±0.17b	2.06±0.12b	2.30±0.13bc	2.26±0.13b
Terpinen-4-ol	33.14	1557	tr c	1.36±0.08ab	1.68±0.10a	1.50±0.09a	1.94±0.11a	1.25±0.07ab	0.99±0.06b	1.36±0.08ab

(E)-2-Octen-1-ol	33.44	1565	0.02±0.00a	0.01±0.00a	0.02±0.00a	0.01±0.00a	0.02±0.00a	tr b	0.02±0.00b	0.04±0.00a
(E)-p-Menth-2-en-1-ol	34.13	1581	tr d	0.38±0.02ab	0.45±0.03a	0.38±0.02ab	0.51±0.03a	0.32±0.02ab	0.27±0.02a	0.40±0.02ab
Myrtenal	34.34	1587	0.12±0.01a	0.10±0.01a	0.12±0.01a	0.10±0.01a	0.12±0.01a	0.09±0.01a	0.07±0.00c	0.12±0.01a
γ-Elemene	34.64	1594	0.15±0.01a	0.06±0.00a	0.14±0.01a	0.15±0.01a	0.07±0.00a	0.06±0.00a	0.06±0.00a	0.08±0.00a
Aromandendrene	34.88	1600	0.18±0.01b	0.16±0.01b	0.17±0.01b	0.16±0.01b	0.13±0.01b	0.09±0.01b	0.28±0.02a	tr c
2,6-Dimethyl-5-hepten-1-ol	35.06	1605	0.06±0.00b	0.05±0.00b	0.07±0.00ab	0.09±0.01a	0.08±0.00a	0.04±0.00b	tr c	0.08±0.00a
Citronellyl acetate	35.49	1617	0.33±0.02a	0.17±0.01b	0.13±0.01bc	0.17±0.01b	0.09±0.00c	0.21±0.01b	0.05±0.00c	0.07±0.00c
(E)-β-Bergamotene	35.67	1621	0.36±0.02a	0.11±0.01b	0.12±0.01b	0.22±0.01ab	0.11±0.01b	0.10±0.01b	0.32±0.02a	0.11±0.01b
Isoborneol	35.72	1623	tr d	0.10±0.01bc	0.13±0.01bc	tr d	0.18±0.01b	0.07±0.00c	0.38±0.02a	0.19±0.01b
(E)-Piperitol	36.17	1635	tr b	0.09±0.01a	0.07±0.00a	0.11±0.01a	0.08±0.00a	0.09±0.01a	0.06±0.00a	0.05±0.00a
Neral	36.46	1642	9.88±0.57a	3.68±0.21c	5.18±0.30b	3.67±0.21c	5.35±0.31b	10.93±0.63a	6.64±0.38b	10.24±0.59a
β-Acoradiene	36.60	1646	tr d	0.08±0.00c	0.32±0.02a	0.09±0.01c	0.08±0.00c	0.45±0.03a	0.08±0.00c	0.24±0.01b
β-Himachalene	36.70	1649	0.44±0.03b	0.46±0.03b	0.20±0.01c	0.52±0.03a	0.22±0.01c	tr d	0.61±0.04a	0.13±0.01c
α-Terpineol	36.91	1654	2.36±0.14a	2.08±0.12a	2.51±0.14a	2.02±0.12a	2.56±0.15a	2.06±0.12a	1.71±0.10a	2.45±0.14a
endo-Borneol	37.07	1659	3.01±0.17a	2.49±0.14a	3.25±0.19a	2.72±0.16a	3.21±0.19a	2.94±0.17a	2.09±0.12a	3.08±0.18a
Germacrene D	37.34	1666	0.07±0.00a	tr b	tr b	tr b	tr b	tr b	tr b	tr b
β-copaene	37.38	1667	tr d	tr d	0.36±0.02b	0.19±0.01c	0.21±0.01c	0.69±0.04a	0.18±0.01c	0.14±0.01c
γ- cadinene	37.51	1671	tr f	tr f	0.12±0.01c	0.50±0.03b	0.15±0.01c	0.02±0.00e	1.11±0.06a	0.07±0.00d
β-copaene +γ- cadinene	37.52	1671	0.87±0.05a	0.44±0.03b	neralral					
α-Funebrene	37.99	1684	4.21±0.24c	9.76±0.56b	10.08±0.58b	12.85±0.74ab	8.74±0.50b	9.32±0.54b	17.34±1.00a	14.28±0.82ab
β-Bisabolene	38.20	1689	4.28±0.25bc	2.38±0.14c	2.21±0.13c	3.13±0.18c	1.71±0.10d	2.56±0.15c	5.90±0.34b	10.20±0.75a
Geranial	38.43	1695	7.16±0.41b	4.51±0.26c	5.66±0.33bc	4.68±0.27c	6.00±0.35bc	12.49±0.72a	3.06±0.18c	2.71±0.12d
(Z)-Piperitol	38.69	1702	tr c	0.11±0.01ab	0.07±0.00b	0.07±0.00b	0.10±0.01ab	0.14±0.01a	0.15±0.01a	0.18±0.01a
(E,E)-α-Farnesene	38.88	1708	3.64±0.21a	2.51±0.15b	1.63±0.09c	2.80±0.16b	1.40±0.08c	1.05±0.06c	4.66±0.27a	2.09±0.12b
(E)-Isopiperitenol	39.03	1712	nd c	0.11±0.01b	0.06±0.00b	0.10±0.01b	0.07±0.00b	tr c	tr c	0.20±0.01a
δ-Cadinene	39.27	1719	0.09±0.01d	0.22±0.01b	0.15±0.01c	0.38±0.02b	0.12±0.01c	0.15±0.01c	0.73±0.04a	0.24±0.01b
Citronellol	39.39	1722	2.18±0.13a	1.92±0.11b	1.94±0.11b	1.94±0.11b	2.40±0.14a	1.32±0.08c	2.49±0.14a	2.21±0.13a
α-Maaliene	39.58	1728	0.08±0.00c	0.10±0.01c	0.01±0.00a	0.22±0.01b	0.02±0.00d	0.25±0.01b	0.43±0.02a	0.18±0.01bc
α-Zingiberene	39.79	1734	5.13±0.30b	5.94±0.34a	4.66±0.27bc	6.51±0.38a	3.99±0.23c	1.91±0.11d	7.01±0.40a	4.18±0.24bc
p-Mentha-1,5-dien-8-ol	40.10	1742	0.11±0.01a	0.10±0.01ab	0.14±0.01a	0.15±0.01a	0.15±0.01a	0.08±0.00b	0.08±0.00b	0.15±0.01a
Myrtenol	40.46	1753	0.14±0.01b	0.10±0.01a	0.10±0.01a	0.10±0.01a	0.11±0.01a	0.08±0.00a	0.08±0.00a	0.13±0.01a
Nerol	40.66	1759	0.20±0.01a	0.11±0.01b	0.12±0.01b	0.13±0.01b	0.13±0.01b	tr c	0.16±0.01a	0.17±0.01a
Isogeraniol	40.96	1767	nd c	nd c	0.07±0.00ab	0.11±0.01a	0.07±0.00ab	0.04±0.00b	0.04±0.00b	0.04±0.00b

Isocarveol	41.97	1796	0.69±0.04a	0.46±0.03a	0.50±0.03a	0.45±0.03a	0.47±0.03a	0.46±0.03a	0.57±0.03a	0.55±0.03a
Geraniol	42.35	1807	1.75±0.10a	1.19±0.07b	1.17±0.07b	0.90±0.05c	1.22±0.07b	0.89±0.05c	1.08±0.06bc	1.38±0.08b
Muurola-4,10(14)-dien-1-β-ol	48.27	1984	tr b	0.11±0.01a	tr b	tr b	tr b	tr b	tr b	tr b
(E)-Nerolidol	49.04	2008	0.40±0.03a	0.12±0.01b	0.04±0.00c	nd d	nd d	nd d	nd d	nd d
10-epi-γ-Eudesmol	51.29	2080	0.03±0.00a	0.05±0.00a	nd b	nd b	nd b	nd b	nd b	nd b
Valerianol	52.15	2107	0.04±0.00a	0.03±0.00a	nd b	nd b	nd b	nd b	nd b	nd b
α-Acorenol	52.28	2111	0.10±0.01a	0.06±0.00a	0.05±0.00b	nd c	nd c	nd c	nd c	nd c
Eugenol	53.48	2150	0.08±0.00a	nd b	nd b	nd b	nd b	nd b	nd b	nd b
Cryptomeridiol	55.22	2207	0.16±0.01a	0.14±0.01a	0.11±0.01a	nd b	nd b	nd b	nd b	nd b

[1] Linear retention index on polar column calculated according to the Van Den Dool and Kratz equation; [2] Percentage of peak area in Total Ion Current (TIC) chromatograms; [3] traces = Inferior to 0.005%. [4] not detected = inferior to LOD. Data are expressed as the mean of three replicates ± SE. Different letters in the same row indicate statistically significant differences by Turkey's multiple range test at $p < 0.05$ during the refrigerated storage time.

Table S3 - Volatile profile composition (%) of Tropical extracts during primary and secondary shelf life under refrigerated storage conditions.

Compounds	RT	LRI	Tro_Ctrl T0_PSL	Tro_Ctrl T33 _PSL = Tro_Ctrl T0_SSL	Tro_Lc T33 _PSL = Tro_Lc T0_SSL	Tro_Lp T33 _PSL = Tro_Lp T0_SSL	Tro_Lr T33 _PSL = Tro_Lr T0_SSL	Tro_Lc T4_SSL	Tro_Lp T4_SSL	Tro_Lr T4_SSL
Ethyl Acetate	6.32	893	1.00±0.06b	1.33±0.08b	2.32±0.13a	2.41±0.14a	2.34±0.14a	0.81±0.05b	1.04±0.06b	0.90±0.05b
Methanol	6.45	899	0.39±0.02a	0.45±0.03a	0.38±0.02a	0.30±0.02a	0.39±0.02a	0.34±0.02a	0.39±0.02a	0.30±0.02a
Methyl propionate	6.68	909	tr c	0.05±0.00b	0.10±0.01a	0.10±0.01a	0.09±0.01a	0.04±0.00b	0.05±0.00b	0.04±0.00b
Ethanol	7.14	928	3.42±0.20cd	4.64±0.27b	5.37±0.31b	6.07±0.35a	7.52±0.43a	2.87±0.17d	3.28±0.19cd	2.65±0.15d
Ethyl propionate	7.64	950	tr b	0.07±0.00a	0.06±0.00a	0.06±0.00a	0.05±0.00a	0.03±0.00a	0.04±0.00a	0.04±0.00a
Propyl acetate	8.12	970	0.07±0.00b	0.08±0.00b	0.13±0.01a	0.13±0.01a	0.12±0.01a	0.07±0.00b	0.10±0.01ab	0.08±0.00b
2-Pentanone	8.22	975	1.35±0.08c	2.16±0.12b	3.57±0.21ab	4.01±0.23a	4.35±0.25a	1.13±0.07c	1.88±0.11c	1.41±0.08c
Methyl butanoate	8.34	980	0.18±0.01b	0.17±0.01b	0.29±0.02a	0.27±0.02a	0.25±0.01a	0.17±0.01b	0.21±0.01ab	0.17±0.01b
Methyl 2-methylbutanoate	8.96	1004	0.42±0.02ab	0.55±0.03a	0.38±0.02b	0.33±0.02b	0.30±0.02b	0.50±0.03a	0.48±0.03a	0.48±0.03a
2-Methylpropyl acetate	9.12	1009	0.80±0.05b	0.85±0.05b	0.88±0.05b	0.77±0.04b	0.73±0.04b	0.89±0.05b	1.17±0.07a	1.11±0.06a
α-Pinene	9.36	1015	0.22±0.01a	0.28±0.02a	tr c	tr c	tr c	0.10±0.01b	0.27±0.02a	0.09±0.01b
3-Pentenone	9.37	1016	nd d	nd d	0.41±0.02a	0.27±0.02b	0.34±0.02ab	0.15±0.01c	tr d	0.19±0.01c
Ethyl butanoate	9.82	1029	0.72±0.04a	0.80±0.05a	0.72±0.04a	0.67±0.04a	0.66±0.04a	0.74±0.04a	0.81±0.05a	0.71±0.04a
Ethyl 2-methylbutanoate	10.31	1042	0.40±0.02ab	0.61±0.04a	0.40±0.02ab	0.40±0.02ab	0.32±0.02b	0.55±0.03a	0.47±0.03ab	0.57±0.03a
Camphene	10.66	1052	0.03±0.00a	nd b	nd b	nd b	nd b	nd b	nd b	nd b
Butyl acetate	10.89	1059	1.72±0.39a	tr b	tr b	tr b	tr b	nd b	nd b	nd b
2-Pentyl acetate	10.99	1062	5.04±0.29c	7.93±0.46a	7.29±0.42ab	7.01±0.40ab	6.87±0.40b	7.71±0.45a	8.68±0.50a	8.13±0.47a
Hexanal	11.33	1071	2.20±0.13c	2.08±0.12c	5.76±0.33a	3.21±0.19bc	4.07±0.24b	3.66±0.21bc	6.45±0.37a	5.25±0.30a
2-Methyl-1-propanol	11.75	1083	nd b	nd b	0.05±0.00a	0.07±0.00a	0.08±0.00a	tr b	tr b	tr b
β-Pinene	11.87	1086	nd c	0.04±0.00b	0.03±0.00b	0.07±0.00a	0.02±0.00b	0.02±0.00b	tr c	0.02±0.00b
2-Methylbutyl acetate	12.54	1104	4.00±0.23bc	2.91±0.17d	4.08±0.24bc	3.84±0.22c	3.17±0.18c	5.32±0.31b	5.57±0.32b	9.96±0.57a
2-Pentanol	12.73	1108	0.06±0.00c	tr d	0.63±0.04a	0.82±0.05a	0.81±0.05a	0.12±0.01b	0.28±0.02b	0.21±0.01b
(E)-2-Pentenal	13.05	1115	tr b	0.10±0.01a	0.11±0.01a	0.08±0.00a	0.09±0.00a	tr b	tr b	tr b
Butyl proprionate	13.32	1121	nd c	nd c	0.08±0.00a	0.07±0.00a	0.07±0.00a	0.02±0.00b	tr c	0.06±0.00a
3-Carene	13.50	1124	11.22±0.65a	9.31±0.54ab	6.96±0.40c	8.59±0.50b	7.46±0.43c	9.23±0.53ab	4.93±0.28d	8.16±0.47b
3-Hexenal	13.52	1125	tr d	tr d	tr d	tr d	tr d	0.12±0.01c	0.35±0.02a	0.28±0.02b
Myrcene	13.72	1129	1.29±0.07a	0.79±0.05b	0.55±0.03cd	0.68±0.04c	0.43±0.02d	0.89±0.05b	0.34±0.02a	0.84±0.05b
β-Pinene	13.90	1133	tr a	tr d	tr d	tr d	tr d	1.05±0.06b	0.81±0.05c	1.61±0.09a
Pseudolimonene (= p-Mentha-1(7),8-diene)	14.09	1137	tr b	0.45±0.03a	0.61±0.04a	0.72±0.04a	0.53±0.03a	tr b	tr b	tr b

1-Butanol	14.15	1138	0.80±0.05c	tr d	1.67±0.10b	1.82±0.10b	2.52±0.15a	0.52±0.03c	0.64±0.04c	tr d
Ethyl (E)-2-butenoate	14.36	1142	nd c	nd c	0.07±0.00ab	0.08±0.00a	0.05±0.00b	0.09±0.01a	0.06±0.00b	0.09±0.01a
α-Terpinene	14.39	1143	nd d	nd d	0.06±0.00b	0.08±0.00a	0.06±0.00b	0.04±0.00c	0.02±0.00c	0.09±0.01a
Pentyl acetate	14.51	1146	0.20±0.01a	0.09±0.00b	0.10±0.01b	0.10±0.01b	0.08±0.00b	0.21±0.01a	0.24±0.01a	0.28±0.02a
2-Heptanone	15.13	1159	1.08±0.06ab	1.13±0.07ab	0.96±0.06b	0.91±0.05b	0.79±0.05b	1.09±0.06ab	1.42±0.08a	1.05±0.06ab
Limonene	15.38	1164	23.14±1.34a	26.37±1.52a	9.25±0.53c	11.32±0.65c	9.83±0.57c	16.78±0.97b	7.49±0.43d	11.17±0.65c
β-Phellandrene	15.67	1170	0.36±0.02a	0.24±0.01ab	tr d	tr d	tr d	0.18±0.01b	0.09±0.01c	0.16±0.01b
2-Pentyl butanoate	15.97	1177	3.28±0.19a	1.75±0.10c	2.16±0.12bc	2.91±0.17b	2.18±0.13bc	3.06±0.18a	2.43±0.14b	3.78±0.22a
Eucalyptol	16.09	1179	0.18±0.01a	0.05±0.00b	tr c	tr c	tr c	tr c	tr c	tr c
Butyl butanoate	16.29	1184	3.00±0.17a	0.91±0.05a	0.85±0.05d	0.96±0.06d	0.75±0.04d	2.24±0.13b	2.14±0.12b	1.79±0.10c
2-Methyl-1-butanol	16.50	1188	tr c	1.36±0.08c	4.12±0.24b	4.31±0.25b	5.16±0.30a	0.89±0.05d	1.31±0.08c	1.21±0.07c
3-Methyl-1-butanol	16.59	1190	tr b	0.38±0.02a	tr b	tr b	tr b	tr b	tr b	tr b
(E)-2-Hexenal	16.79	1194	3.30±0.19d	2.40±0.14d	10.82±0.62a	5.99±0.35c	7.29±0.42b	7.37±0.43b	11.81±0.68a	7.38±0.43b
Butyl 2-methylbutanoate	16.99	1198	0.20±0.01b	tr c	tr c	tr c	tr c	0.35±0.02a	0.28±0.02ab	0.27±0.02ab
γ-Terpinene	17.35	1206	0.08±0.00ab	0.09±0.01ab	0.14±0.01a	0.11±0.01ab	0.14±0.01a	0.07±0.00ab	0.05±0.00b	0.10±0.01ab
2-Pentyl pentanoate	17.50	1209	0.15±0.01a	0.11±0.01a	0.15±0.01a	0.18±0.01a	0.19±0.01a	0.15±0.01a	0.11±0.01a	0.18±0.01a
β-Ocimene	17.62	1212	0.84±0.05a	0.71±0.04a	0.41±0.02ab	0.43±0.02ab	0.50±0.03ab	0.70±0.04a	0.34±0.02b	0.70±0.04a
Butyl 3-methylbutanoate	17.81	1216	tr b	tr b	0.09±0.01a	0.08±0.00a	0.10±0.01c	tr b	tr b	tr b
5-Methyl-5-hexen-2-one	18.14	1223	0.65±0.04a	0.81±0.05a	0.91±0.05a	0.80±0.05a	0.73±0.04a	0.80±0.05a	1.15±0.07a	0.63±0.04a
3-Methylbutyl butanoate	18.32	1227	5.00±0.29a	2.74±0.16c	2.93±0.17c	3.73±0.22b	2.96±0.17c	5.13±0.30a	3.76±0.22b	5.24±0.30a
p-Cymene	18.57	1233	0.09±0.01a	0.06±0.00a	0.06±0.00a	0.06±0.00a	0.10±0.01a	0.06±0.00a	0.06±0.00a	0.08±0.00a
Hexyl acetate	18.79	1237	1.58±0.09a	0.33±0.02c	0.23±0.01c	0.23±0.01c	0.17±0.01c	1.55±0.09a	1.06±0.06b	1.18±0.07b
Terpinolene	19.03	1242	tr c	0.96±0.06a	0.39±0.02b	0.51±0.03b	0.42±0.02b	0.73±0.04ab	0.41±0.02b	0.66±0.04ab
3-Methylbutyl 2-methylbutanoate	19.07	1243	0.12±0.01a	tr c	0.15±0.01a	0.19±0.01a	0.15±0.01a	0.16±0.01a	0.04±0.00b	0.19±0.01a
3-Methylbutyl 3-methylbutanoate	19.55	1254	2.57±0.15a	1.43±0.08b	1.65±0.10b	2.09±0.12a	1.81±0.10b	2.42±0.14a	1.72±0.10b	2.67±0.15a
2-Heptanol	21.06	1286	0.35±0.02a	0.41±0.02a	0.56±0.03a	0.53±0.03a	0.49±0.03a	0.36±0.02a	0.60±0.03a	0.41±0.02a
(Z)-2-Penten-1-ol	21.25	1290	tr c	0.04±0.00b	0.09±0.01a	0.10±0.01c	0.13±0.01a	tr c	tr c	tr c
(Z)-2-Heptenal	21.35	1292	nd d	0.05±0.00c	0.07±0.00c	0.13±0.01b	0.07±0.00c	0.14±0.01b	0.24±0.01a	0.24±0.01a
6-Methyl-5-hepten-2-one	21.86	1303	0.10±0.01ab	0.10±0.01ab	0.05±0.00b	0.10±0.01ab	0.06±0.00b	0.14±0.01a	0.17±0.01a	0.14±0.01a
4-Methyl-5-hexen-2-ol	22.15	1309	0.40±0.02b	0.57±0.03b	0.70±0.04a	0.83±0.05a	0.79±0.05a	0.50±0.03b	0.71±0.04a	0.51±0.03b
Hexanol	22.57	1318	7.18±0.41c	9.68±0.56b	11.34±0.65a	11.27±0.65a	12.19±0.70a	7.40±0.43c	10.13±0.58ab	6.01±0.35c
(E)-3-Hexen-1-ol	23.02	1327	nd b	0.05±0.00a	0.06±0.00a	0.05±0.00a	0.07±0.00a	0.03±0.00a	0.04±0.00a	0.03±0.00a

(Z)-3-Hexen-1-ol	23.93	1346	0.11±0.01b	0.26±0.02a	0.22±0.01a	0.19±0.01ab	0.23±0.01a	0.11±0.01b	0.16±0.01ab	0.09±0.01b
2-Nonanone	24.18	1351	nd c	nd c	0.04±0.00b	0.05±0.00b	0.04±0.00b	0.09±0.01a	0.07±0.00ab	0.11±0.01a
Nonanal	24.28	1353	nd d	nd d	0.10±0.01c	0.23±0.01a	0.18±0.01b	0.06±0.00c	0.04±0.00c	0.09±0.01c
2-Methylpropyl hexanoate	24.30	1354	nd d	0.50±0.03a	0.17±0.01c	0.30±0.02b	0.32±0.02b	0.30±0.02b	0.27±0.02b	0.28±0.02b
3-Pentyl hexanoate	24.36	1355	tr b	0.04±0.00a	0.02±0.00a	0.05±0.00a	0.03±0.00a	0.03±0.00a	0.02±0.00a	tr b
(Z)-2-Hexen-1-ol	24.85	1365	1.93±0.11c	2.72±0.16b	3.14±0.18a	2.61±0.15b	3.54±0.20a	1.99±0.11c	2.60±0.15b	1.54±0.09c
Hexyl butanoate	25.12	1370	0.39±0.02a	0.18±0.01c	0.06±0.00a	0.07±0.00d	0.06±0.00a	0.27±0.02b	0.21±0.01b	0.22±0.01b
4-Methylpentyl 2-methylbutanoate	25.58	1380	tr c	tr c	tr c	tr c	tr c	0.16±0.01a	0.09±0.01b	0.11±0.01ab
(E)-2-Octenal	26.00	1388	nd b	nd b	nd b	nd b	nd b	0.10±0.01a	0.12±0.01a	0.12±0.01a
Hexyl 3-methylbutanoate	26.30	1395	0.15±0.01b	0.19±0.01b	0.09±0.00c	0.01±0.00d	0.12±0.01b	0.29±0.02a	0.27±0.02a	0.30±0.02a
2-Methylcyclohexyl trans-butanoate	26.48	1398	tr d	0.44±0.03b	0.36±0.02c	0.45±0.03b	0.44±0.03b	0.55±0.03a	0.47±0.03b	0.65±0.04a
1-Octen-3-ol	26.65	1402	0.09±0.01b	0.04±0.00c	0.10±0.01b	0.08±0.00b	0.09±0.01b	0.15±0.01a	0.19±0.01a	0.12±0.01ab
3-Methylbutyl hexanoate	26.90	1408	0.16±0.01b	0.26±0.02a	tr d	tr d	0.07±0.00c	0.17±0.01b	0.11±0.01c	0.10±0.01c
Acetic acid	27.11	1413	1.00±0.06a	1.11±0.06a	0.31±0.02b	0.38±0.02b	0.12±0.01c	0.34±0.02b	1.09±0.06a	0.87±0.05a
2-Methylcyclohexyl trans-pentanoate	27.70	1427	nd c	0.14±0.01a	0.04±0.00b	0.05±0.00b	0.06±0.00b	0.09±0.01ab	0.07±0.00b	0.09±0.00ab
2-Ethyl-1-hexanol	28.36	1442	0.46±0.03a	0.42±0.02a	0.06±0.00d	0.03±0.00d	0.04±0.00d	0.14±0.01bc	0.11±0.01c	0.20±0.01b
α-Copaene	28.40	1443	0.18±0.01b	tr d	0.04±0.00c	0.09±0.01c	0.06±0.00c	0.23±0.01a	0.23±0.01a	0.25±0.01a
Decanale	28.83	1453	nd b	nd b	nd b	nd b	nd b	0.13±0.01a	0.16±0.01a	0.18±0.01a
Methyl 3-(methylthio)propionate	30.01	1481	0.40±0.02a	0.16±0.01c	tr d	tr d	tr d	0.28±0.02b	0.21±0.01b	0.10±0.01c
Benzaldehyde	30.19	1485	0.76±0.04c	1.70±0.10a	0.91±0.05b	1.27±0.07ab	1.10±0.06b	0.89±0.05b	1.43±0.08a	1.03±0.06b
Linalool	30.75	1498	0.73±0.04a	0.46±0.03ab	0.20±0.01b	0.30±0.02b	0.21±0.01b	0.70±0.04a	0.74±0.04a	0.56±0.03ab
1-Octanol	31.19	1509	0.43±0.02a	0.26±0.01bc	0.17±0.01c	0.18±0.01c	0.10±0.01d	0.35±0.02b	0.38±0.02b	0.26±0.01bc
2,5-Dimethyl-4-methoxy-3(2H)-furanone	32.82	1549	tr c	0.35±0.02a	0.14±0.01b	0.16±0.01b	0.14±0.01b	tr c	tr c	tr c
Caryophyllene	32.89	1551	1.10±0.06a	0.46±0.03b	0.45±0.03b	0.28±0.02c	0.33±0.02bc	0.95±0.06a	1.04±0.06a	0.68±0.04b
Terpinen-4-ol	33.14	1557	0.13±0.01a	0.10±0.01a	0.08±0.00a	0.10±0.01a	0.11±0.01a	0.10±0.01c	0.12±0.01a	0.05±0.00b
Hexyl hexanoate	33.29	1561	0.26±0.02a	0.09±0.01c	tr d	tr d	tr d	0.17±0.01b	0.16±0.01b	0.09±0.01c
(E)-2-Octen-1-ol	33.44	1565	0.06±0.00a	tr b	tr b	tr b	tr b	tr b	tr b	tr b
Butanoic acid	34.30	1586	0.72±0.04b	0.78±0.05b	0.27±0.02c	0.35±0.02c	0.21±0.01c	0.47±0.03bc	0.93±0.05a	0.49±0.03bc
α-Humulene	35.80	1625	tr d	0.21±0.01b	0.34±0.02b	0.25±0.01b	0.54±0.03a	0.02±0.00c	0.02±0.00c	0.04±0.00c
α-Terpineol	36.91	1654	0.29±0.02b	0.22±0.01b	0.24±0.01b	0.24±0.01b	0.22±0.01b	0.35±0.02ab	0.43±0.02a	0.35±0.02ab
Geranial	38.43	1695	0.19±0.01a	0.08±0.00b	0.09±0.01b	0.11±0.01b	0.07±0.00b	0.11±0.01b	0.18±0.01a	0.20±0.01a

(E,E)-α-Farnesene	38.88	1708	0.18±0.01a	tr b	tr b	tr b	tr b	tr b	tr b	tr b
Hexanoic acid	42.38	1808	1.12±0.06a	0.27±0.02d	0.73±0.04b	0.94±0.05ab	0.42±0.02c	0.45±0.03c	1.08±0.06a	0.85±0.05ab
Octanoic acid	49.73	2030	nd d	nd d	0.25±0.01b	0.27±0.02b	0.23±0.01b	0.07±0.00c	0.34±0.02a	0.05±0.00c
Eugenol	53.48	2150	tr d	0.13±0.01c	0.16±0.01c	0.10±0.01c	0.13±0.01c	0.21±0.01b	0.52±0.03a	0.42±0.02a

[1] Linear retention index on polar column calculated according to the Van Den Dool and Kratz equation; [2] Percentage of peak area in Total Ion Current (TIC) chromatograms; [3] traces = Inferior to 0.005%. [4] not detected = inferior to LOD. Data are expressed as the mean of three replicates ± SE. Different letters in the same row indicate statistically significant differences by Turkey's multiple range test at $p < 0.05$ during the refrigerated storage time.
